# Adipokines and their potential impacts on susceptibility to myocardial ischemia/reperfusion injury in diabetes

**DOI:** 10.1186/s12944-024-02357-w

**Published:** 2024-11-13

**Authors:** Ronghui Han, Hemeng Huang, Jianyu Zhu, Xiaogao Jin, Yongyan Wang, Youhua Xu, Zhengyuan Xia

**Affiliations:** 1 Faculty of Chinese Medicine, State Key Laboratory of Quality Research in Chinese Medicine, Macau University of Science and Technology, Avenida Wai Long, Taipa, Macao, PR China; 2 Department of Anesthesiology, Affiliated Hospital of Guangdong Medical University, Guangdong, China; 3 Department of Emergency, Affiliated Hospital of Guangdong Medical University, Guangdong, China; 4 Department of Anesthesiology, The Second Affiliated Hospital of Guangdong Medical University, Guangdong, China; 5 Macau University of Science and Technology Zhuhai MUST Science and Technology Research Institute, Hengqin, Zhuhai, People’s Republic of China; 6 Faculty of Pharmacy, Macau University of Science and Technology, Avenida Wai Long, Taipa, Macao, People’s Republic of China; 7 State Key Laboratory of Pharmaceutical Biotechnology, Department of Medicine, The University of Hong Kong, Hong Kong, China

**Keywords:** Diabetes, Myocardial ischemia/reperfusion (I/R) injury, Adipokines

## Abstract

Coronary artery disease has a high mortality rate and is a striking public health concern, affecting a substantial portion of the global population. On the early onset of myocardial ischemia, thrombolytic therapy and coronary revascularization could promptly restore the bloodstream and nutrient supply to the ischemic tissue, efficiently preserving less severely injured myocardium. However, the abrupt re-establishment of blood flow triggers the significant discharge of previously accumulated oxidative substances and inflammatory cytokines, leading to further harm referred to as ischemia/reperfusion (I/R) injury. Diabetes significantly raises the vulnerability of the heart to I/R injury due to disrupted glucose and lipid processing, impaired insulin sensitivity and metabolic signaling, and increased inflammatory responses. Numerous studies have indicated that adipokines are crucial in the etiology and pathogenesis of obesity, diabetes, hyperlipidemia, hypertension, and coronary artery disease. Adipokines such as adiponectin, adipsin, visfatin, chemerin, omentin, and apelin, which possess protective properties against inflammatory activity and insulin resistance, have been shown to confer myocardial protection in conditions such as atherosclerosis, myocardial hypertrophy, myocardial I/R injury, and diabetic complications. On the other hand, adipokines such as leptin and resistin, known for their pro-inflammatory characteristics, have been linked to elevated cardiac lipid deposition, insulin resistance, and fibrosis. Meteorin-like (metrnl) exhibits opposite effects in various pathological conditions. However, the data on adipokines in myocardial I/R, especially in diabetes, is still incomplete and controversial. This review focuses on recent research regarding the categorization and function of adipokines in the heart muscle, and the identification of different signaling pathways involved in myocardial I/R injury under diabetic conditions, aiming to facilitate the exploration of therapeutic strategies against myocardial I/R injury in diabetes.

**Supplementary Information:**

The online version contains supplementary material available at 10.1186/s12944-024-02357-w.

## Introduction

As claimed by the most recent American Heart Association report, mortality rates stemming from cardiac and cardiovascular disease (CVD) have declined by 60% since 1950 as a result of precise diagnostic techniques and proactive medical and surgical interventions in the United States [1]. Coronary artery bypass grafting off or on-pump and direct percutaneous coronary intervention are widely recognized as the most efficacious therapeutic modalities for myocardial preservation in instances of cardiac injury [2, 3]. It is essential to understand that the sudden reintroduction of oxygen and nutrients can disturb the function and electrical activity of the heart muscle, leading to damage and exacerbating myocardial necrosis, a condition referred to as myocardial ischemia/reperfusion (I/R) injury [4]. In light of recent advancements in research on the molecular mechanisms of myocardial I/R injury, significant attention has been paid to elucidating the role of mitochondrial, lipid and glucose metabolism, oxidative products, calcium regulation, and cell signaling [5, 6]. However, there remains a lack of effective treatments for this condition in medical settings.

Conversely, there has been a slow increase in mortality rates from the end of the 2010s to 2020, which is due to the worsening of risk hazards such as diabetes, hypertension, obesity, aging population, and other related factors [1]. What is more, the global prevalence of diabetes reached 529 million individuals in 2021, with projections indicating a significant increase to 1.31 billion by the year 2050 [7]. Recent research indicates that diabetes can heighten the heart's responsiveness to I/R damage, reduce the heart's ability to respond to protective measures, disrupt energy metabolism, worsen the oxidative response and inflammatory activity in the heart, and consequently raise the likelihood of cardiomyocyte death through various mechanisms like apoptosis, necroptosis, ferroptosis, and pyroptosis [8, 9]. It is crucial to comprehend the pathogenic process that controls the advancement and worsening of myocardial I/R injury under hyperglycemia and to investigate reliable cardiac biomarkers for predicting risk.

Previously considered solely as a non-active energy receiver, white adipose tissue (WAT) has recently been acknowledged as an essential endocrine component that generates multiple peptide hormones with autocrine, paracrine, or endocrine effects on diverse physiological processes [10, 11]. These secretions comprise a varied assortment of small chemical molecules, such as cytokines and chemokines, that engage with adipose cells, immune cells, and non-regenerative cells (osteoblasts, neurocytes, retinal cells, pancreatic β cells, and cardiomyocytes) [12]. Certain agents are generated by cells other than adipocytes. In contrast, others are secreted by adipocytes and categorized as adipokines that include but are not limited to adiponectin, leptin, resistin, apelin, adipsin, visfatin, omentin, chemerin and meteorin-like (metrnl) [13]. Recent research has demonstrated that adipokines have a multifaceted impact on the insulin sensitivity, atherosclerosis, inflammation, and myocardial signaling pathogenesis. They display seemingly contradictory effects on the heart's functionality, particularly after oxidative products and I/R injury [14–16]. In a comprehensive analysis of preclinical animal studies, it was observed that adiponectin, possessing insulin-sensitizing and anti-inflammatory attributes, effectively suppressed apoptosis in cardiac muscle cells exposed to reperfusion injury by stimulating diverse molecular pathway cascades [17]. For instance, a study proposed that administering adiponectin as a supplement could potentially strengthen the responsiveness of the diabetic heart to ischemic post-conditioning through the initiation of diverse cellular signaling pathways, including Janus-activated kinase (JAK) /signal transducers and activators of transcription 3 (STAT3) and AMP-activated protein kinase (AMPK) [18]. Besides, intracerebroventricular administration of leptin significantly mitigated cardiac malfunction following I/R injury, as proved by enhanced ventricular systolic function, overall cardiac function, and mitochondrial metabolism [19]. Therefore, evidence suggests that adipokines are vital in developing myocardial I/R injury in subjects with or without diabetes, but its molecular mechanism remains largely unclear.

Previous research has mainly concentrated on individual adipokines or their receptors in relation to myocardial I/R injury or diabetes, highlighting the significant impact of adipokines on cardiovascular disease through glucose and lipid metabolism disorders [20–22]. By contrast, this review mainly examined the regulation and effects of different adipokines and associated signaling pathways and discussed their potential impacts on myocardial I/R injuries. Furthermore, a network analysis was conducted on various adipokines and their corresponding receptors using STRING tools and Cytoscape software. The aim was to delineate potential interactions among adipokines in the context of myocardial I/R injury in individuals with diabetes. This comprehensive research was undertaken to facilitate the development of innovative therapeutics and preventive strategies.

## Increased vulnerability to myocardial I/R injury in diabetes

Diabetes, a metabolic disorder, is marked by high blood sugar levels resulting from insufficient insulin secretion or activity. It affects a substantial portion of the worldwide populace and is associated with various complications, particularly cardiovascular disease [23]. Of these complications, myocardial I/R injury is a prominent issue. Tissue damage arises as a consequence of the bloodstream supply briefly halted and then restored. Diabetic individuals are more sensitive to myocardial I/R injury, and the mechanisms driving this are discussed in the following sections.

### Clinical perspective

Numerous studies have consistently shown that individuals with diabetes exhibit a heightened sensitivity to myocardial I/R injury when compared to non-diabetic counterparts [24, 25]. This increased risk is attributed to various underlying pathophysiological mechanisms, including hyperglycemia/hyperlipidemia, insulin resistance, compromised coronary microcirculation, endothelial dysfunction, elevated oxidative products, and dysregulated inflammatory activities [26]. Hyperglycemia is associated with an imbalanced generation of reactive oxygen species (ROS) and antioxidant defense malfunction, resulting in oxidative stress and exacerbating myocardial damage during periods of ischemia and reperfusion [27]. Studies from the same laboratory illustrated that elevated glucose levels increased myocardial infarct area during I/R in Mus musculus and enhanced ROS generation in vivo and in vitro [28, 29]. Insulin resistance, a prevalent characteristic of diabetes, hinders the uptake and utilization of glucose in cardiomyocytes, resulting in disrupted energy metabolism, mitochondrial dysfunction, and heightened susceptibility to myocardial injury [30]. Meanwhile, insulin resistance exacerbates inflammation and endothelial dysfunction, exacerbating myocardial dysfunction and augmenting susceptibility to I/R injury [31]. Diabetes-related impairment of angiogenesis impedes the development of new blood vessels in the ischemic myocardium, leading to reduced oxygen and nutrient delivery to the affected region, thereby exacerbating myocardial injury during reperfusion [32]. Additionally, endothelial dysfunction and microvascular abnormalities induced by diabetes contribute to impaired coronary flow reserve, thereby restricting the myocardium's capacity to withstand ischemic insults [33, 34]. There is compelling evidence indicating that healthy platelets possess cardioprotective properties; however, studies have shown that platelets obtained from sufferers with poorly managed type 2 diabetes mellitus (T2DM) exhibit diminished beneficial properties compared to platelets from healthy individuals [35]. Activated platelets migrate into the damaged cardiac muscle and provoke I/R injury by forming micro-emboli-small clots, enhancing platelet-leukocyte aggregation, and releasing vasoconstrictor and pro-inflammatory mediators [36]. Lastly, diabetic patients frequently present with comorbid conditions such as hypertension and dyslipidemia, which further increase their vulnerability to myocardial I/R injury [37]. Effective management of diabetes and its related cardiovascular complications is essential in minimizing the risk of myocardial I/R injury. Maintaining tight control over blood sugar levels (lifestyle changes, oral hypoglycemic medications, or insulin therapy) has been demonstrated to decrease ROS generation, inflammation, and endothelial dysfunction, ultimately reducing the area of cardiac damage during I/R [38]. Nevertheless, it is essential to acknowledge that strict glucose control may have constraints, as evidenced by previous studies demonstrating an elevated risk of cardiac attack in patients undergoing specific anti-hyperglycemic drug therapies [39], and severe hyperglycemia was more common in the strict-glucose-control group [40]. One possible explanation for this phenomenon might be the crucial character of myocardial glucose uptake and metabolism in sustaining myocardial energetic during periods of stress [41, 42].

### Underlying molecular mechanisms of increased vulnerability to myocardial I/R injury in diabetes

**Fig. 1:**
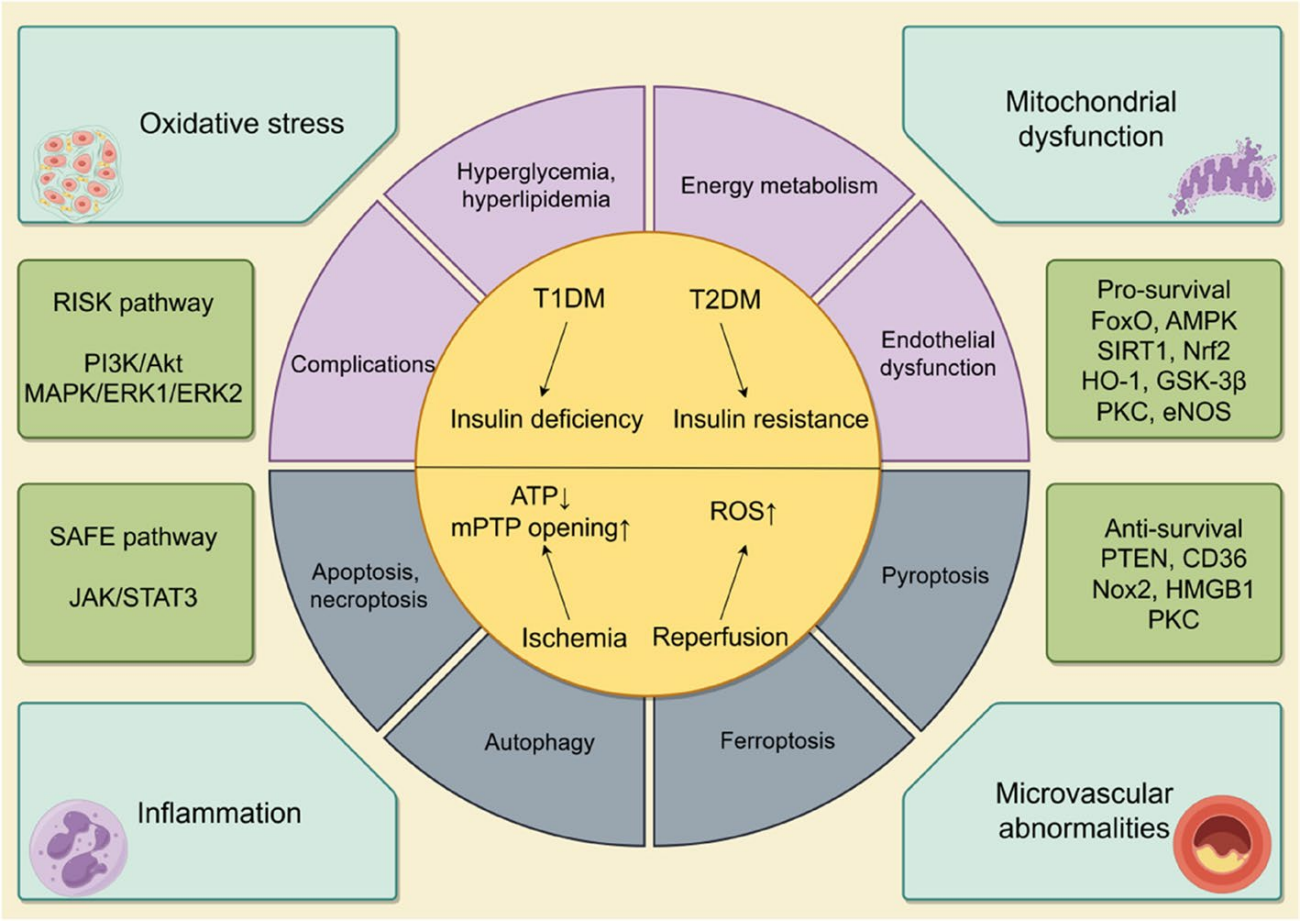
Mechanism of diabetes mellitus aggravating myocardial ischemia/reperfusion (I/R) injury. Diabetes mellitus is categorized predominantly into type 1 diabetes mellitus (T1DM), distinguished by insufficient insulin production, and type 2 diabetes mellitus (T2DM), distinguished by reduced sensitivity to insulin. Diabetic condition primarily leads to hyperglycemia, hyperlipidemia, disrupted energy metabolism, endothelial dysfunction, and a range of associated complications. Under situation of myocardial ischemia, decreased ATP levels, increased opening of the mitochondrial permeability transition pore (mPTP), and the subsequent burst of ROS during myocardial reperfusion contribute to diverse types of cell deaths (including pyroptosis, necrosis, autophagy, ferroptosis, and apoptosis). Diabetes exacerbates myocardial I/R injury primarily through mechanisms involving inflammatory response, oxidative stress, disrupted mitochondrial and microvascular function and the key signal transduction pathways indicated in this action are the RISK and SAFE pathways. Current research indicates that the predominant pro-survival signaling involved are FoxO, AMPK, SIRT1, Nrf2, HO-1, GSK-3β and eNOS and the anti-survival signaling are primarily PTEN, CD36, HMGB1 and Nox2, while PKC exhibits dual function. FoxO: the class O of Forkhead box 1; AMPK: AMP-activated protein kinase; SIRT1:sirtuin 1; Nrf2: nuclear factor E2-related factor 2; HO-1: heme oxygenase-1; GSK-3β: glycogen synthase kinase-3β; eNOS: endothelial nitric oxide synthase; PTEN: Phosphatase and Tensin Homolog; CD36:cluster of differentiation 36; HMGB1: high mobility group box1protein; Nox2: Nicotinamide adenine dinucleotide phosphate (NADPH) oxidase-2;PKC:protein kinase C; ERK 1: extracellular signal-regulated kinase 1; ERK2: extracellular signal-regulated kinase 2; JAK: janus-activated kinase; STAT3: signal transducers and activators of transcription 3; PI3K:phosphoinositide 3-kinase; Akt: protein kinase B; MAPK: mitogen-activated protein kinase

The increased vulnerability to myocardial I/R injury in diabetes to myocardial I/R injury in individuals with diabetes is a multifaceted phenomenon that is impacted by a variety of pathophysiological mechanisms. In other words, it is imperative to comprehend these underlying molecular mechanisms in order to formulate precise therapeutic approaches to mitigate myocardial damage and to enhance clinical outcomes in diabetic populations. The fundamental mechanisms of diabetes aggravating myocardial I/R injury are summarized in the Fig. 1.

Research indicates that the diabetic heart may resist cardioprotective interventions. This could be attributed to disruptions in various signaling pathways and abnormal cardiomyocyte death, as observed in animal studies and in in vitro experiments, although clinical confirmation is currently insufficient [43]. The Reperfusion Injury Signaling Kinase (RISK) pathway, which encompasses the phosphoinositide 3-kinase (PI3K) /protein kinase B (Akt) and extracellular signal-regulated kinase 1 (ERK1) /extracellular signal-regulated kinase 2 (ERK2), mitogen-activated protein kinases (MAPK) signaling pathway, and the Survivor Activating Factor Enhancement (SAFE) pathway, which includes the JAK/STAT3 signaling cascade, play crucial roles in myocardial protection [44]. Impairments in above-mentioned signaling transducers diminish the responsiveness of diabetic myocardium to therapeutic interventions. Many studies have explored the cardiac protective effect of RISK and SAFE during the reperfusion period after the lethal ischemic insult. In this regard, previous findings indicated that activation of these two pathways following the onset of diabetes effectively reduced the size of infarct area caused by I/R and cell death triggered by high glucose and hypoxia/reoxygenation [45]. Further investigation demonstrated that the specific suppression of Phosphatase and Tensin Homolog (PTEN) using the inhibitor bisperoxovanadium sustained the cardioprotective benefits of post-ischemic-conditioning in streptozotocin-induced diabetic Rattus norvegicus by reactivating the PI3K/Akt and Janus-activated kinase 2 (JAK2)/STAT3 pathways [46], suggesting that the heart may regulate the phosphorylation of these kinases through the activation of PTEN under diabetic conditions. However, whether these two pathways may interact or be totally independent during myocardial I/R remains unclear. It appears that STAT5, as opposed to STAT3, may be a significant factor in the process of cardioprotection in the context of human physiology [47, 48]. Additionally, both the class O of Forkhead box (FoxO) transcription factors and the cluster of differentiation 36 (CD36)/AMPK signaling are involved in interventions combating against myocardial I/R injury via inhibiting excessive apoptotic cells, autophagy and ferroptosis as have been described in some recent studies [25, 26]. Recently, ferroptosis has emerged as an unique form of iron-dependent necrosis that differs from apoptosis, autophagy, and other established mechanisms of cell death. To date, several studies have validated the presence of myocardial cell ferroptosis in diabetic animal models, evidenced by the facts that administration of the ferroptosis agonist Erastin exacerbated myocardial I/R injury and post-ischemic cell death while the ferroptosis inhibitor Ferrostatin-1 or the utilization of the antioxidant N-acetylcysteine has been shown to attenuate myocardial I/R injury under high glucose condition [49–51]. AMPK, protein kinase C (PKC), ERK1/2, PI3K, and Akt defend against ferroptosis in myocardial tissue [52], while phosphoenolpyruvate carboxykinase-α/β (PCKα/β) inhibition also yield cardioprotective effects under high glucose [53]. In addition to the signaling mentioned above pathways and programmed cell death mechanisms, a variety of intracellular signaling molecules and cell death processes can also trigger myocardial I/R injury when hyperglycemia is present, such as necroptosis induced by necrosis-related proteins (Caspase 3, Bax, p-RIP3, p-RIP1, and p-MLKL) through the JAK2/STAT3 pathway [54], pyroptosis induced by the NLRP3 inflammasome via AMPK-Nicotinamide adenine dinucleotide phosphate (NADPH) oxidase-2 signaling [55], autophagy regulated by high mobility group box1protein (HMGB1) [56], sirtuin 1 (SIRT1) /nuclear factor E2-related factor 2 (Nrf2)/heme oxygenase-1 (HO-1) pathway activated by exaggerated generation of ROS and poor mitochondrial function [57], endothelial and vascular dysfunction with the suppression of endothelial nitric oxide synthase (eNOS) [58], inhibition of ROS-induced apoptotic rate by activating the AMPK/Akt/GSK-3β (glycogen synthase kinase-3β signaling) and Nrf2-governed antioxidant enzymes activity [59].

Impacts of adipokines on diabetes complicated with myocardial I/R injury.

**Fig. 2:**
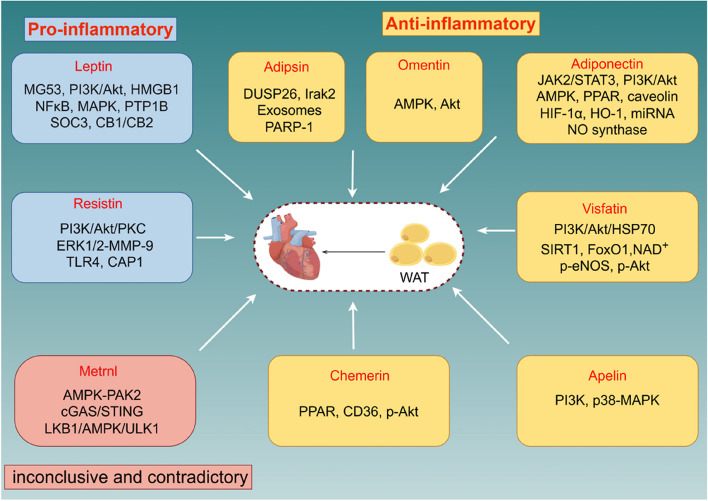
The function and main molecular mechanism of various adipokines on myocardial ischemia/reperfusion (I/R) injury. Adipokines are mainly divided into two categories: anti-inflammatory (including adiponectin, adipsin, visfatin, chemerin, omentin and apelin, related signaling pathways are marked in each corresponding yellow box) and pro-inflammatory (leptin and resistin, related signaling pathways are marked in each corresponding blue box), while metrnl (related signaling pathway are marked in red box) exhibit opposite effects in various pathological condition. WAT: white adipose tissue; JAK2: janus-activated kinase 2; STAT3: signal transducers and activators of transcription 3; PI3K:phosphoinositide 3-kinase; Akt: protein kinase B; AMPK: AMP-activated protein kinase; PPAR: peroxisome proliferator-activated receptor; HIF-1α: hypoxia-inducible factor-1α; HO-1: heme oxygenase-1; miRNA: microRNA; NO synthase: nitric oxide synthase; HSP70: heat shock protein 70; SIRT1: sirtuin 1; FoxO1: the class O of Forkhead box 1; NAD.^+^: nicotinamide adenine dinucleotide; p-eNOS: phosphorylation of endothelial nitric oxide synthase; p-Akt: phosphorylation of protein kinase B; p38/MAPK: p38 mitogen-activated protein kinase; CD36: cluster of differentiation 36; PAK2: serine/threonine protein kinase 2; cGAS-STING: Cyclic GMP-AMP Synthase-Stimulator of interferon genes; LKB1: Liver Kinase B1; ULK1: UNC-51LikeAutophagyAckingKinase1; PKC: protein kinase C; ERK 1: extracellular signal-regulated kinase 1; ERK2: extracellular signal-regulated kinase 2; MMP-9: matrix metalloproteinase 9; TLR4: toll-like receptor 4; CAP1: adenylyl cyclase-associated protein 1; MG53: mitsugumin 53; HMGB1: high mobility group box1protein; NFκB: Nuclear Factor kappa B; MAPK: mitogen-activated protein kinase; PTP1B: protein tyrosine phosphatase 1B; SOC3: suppressor of cytokine signaling 3; CB1: Cannabinoid Receptor 1; CB2: Cannabinoid Receptor 2

Various hormone-like molecules (adiponectin, leptin, resistin, apelin, visfatin, adipsin, omentin, chemerin, and metrnl) are classified as adipokines that are produced by WAT and play a multifaceted role in numerous diseases, including diabetes, atherosclerosis, CVD, and immune disorders [60, 61]. In obese pre-diabetic patients, abdominal fat tissue is a significant source of inflammatory and oxidative stress metabolites. Previous study found that high levels of these molecules are linked to low SIRT1 expression in adipose tissue, potentially impacting heart function both locally and systemically [62]. Furthermore, epicardial fatty tissue has been pinpointed as a leading source of CVD and metabolic disorders after an acute coronary event and coronary inflammation in T2DM given that it is closely adjacent to the coronary arteries [63, 64]. Besides, women have unique atypical risk factors that are associated with the prognosis of CVDs, such as pre-menopausal breast fat accumulation [65]. Adipokines have been implicated as playing both protective and deleterious effects of I/R injury by influencing various molecular pathways (Fig. 2). Myocardial I/R injury and diabetes are both associated with several mechanistic aspects of adipokines that have yet to be clarified.

### Adiponectin

An independent research study employing various experimental methodologies initially characterized adiponectin, a protein uniquely generated by adipose cell and regulated by the ADIPOQ gene, known by various aliases including GBP-28, AdipoM1, AdipoQ, and Acrp30 in humans [66–69]. It typically exhibits high levels in the bloodstream, ranging from 3-30μg/mL, and comprises only 0.01% of pure protein in plasma, in comparison to else hormones and cytokines [70]. Meanwhile, adiponectin contains 247 amino acids including signal sequence, unstable domain, collagen-like domain, globular domain and it subsists in trimers (the fundamental unit), consists of hexamers with low molecular weights and isoforms with high molecular weights [71]. Until now, there are three central adiponectin receptors, including adiponectin receptor 1 (AdipoR1), adiponectin receptor 2 (AdipoR2) and T-cadherin (CDH13). AdipoR1 demonstrates a strong binding affinity towards globular adiponectin, whereas its interaction with full-length adiponectin is relatively weak. On the other hand, adiponectin with globular or full-length exhibits a moderate adaptability for AdipoR2 [72]. In terms of CDH13, it has been regarded as a receptor for hexameric and high molecular weights of isoforms of adiponectin [73].

Plenty of clinical trials have implied that individuals with elevated adiponectin levels have less chance of diabetes and are less susceptible to CVD [74, 75]. Meanwhile, animal experiments have indicated that insulin resistance is improved noticeably and post-ischemic myocardial infarction reduced significantly with exogenous adiponectin or adiponectin receptors agonist [76, 77]. For instance, previous studies have reported that cardiomyocytes from adiponectin knockout Mus musculus sustained severer I/R injury which could not be reverted or ameliorated by peroxisome proliferator-activated receptor (PPAR)-γ or PPAR-γ agonist [78–80]. Furthermore, cardiac adiponectin could act as an adjuster via AdipoR2 to prevent diabetic myocardial I/R injury through PI3K/Akt and JAK2/STAT3 [36, 81]. Furthermore, Cao et al. illustrated that ischemic postconditioning contributed to a significant loss in post-ischemic myocardial infarction and ROS accumulation in normal Rattus norvegicus, a phenomenon closely linked to increased expression of adiponectin and phosphorylated protein kinase B (p-Akt). The above conducive outcomes were canceled in diabetic Rattus norvegicus and the expressions of adiponectin were restrained [82]. Moreover, Li et al. proposed that the protective consequences of ischemic postconditioning are compromised in diabetes as a result of impaired adiponectin/AdipoR1/caveolin-3 signaling [83]. It is noted that caveolin has been generally identified as a tremendous latent spot in multifarious biological processes, including adiponectin signalosome formation and cardiac protection [84]. Wang et al. have shown that the knockout of caveolin-3 significantly blunts adiponectin's anti-apoptotic effect and exacerbates myocardial I/R injury. AdipoR1 co-localizes with caveolin-3 to form a complex, and the latter activates adiponectin cardioprotection signaling pathways, which are regulated by AMPK or not [85]. Recently, a study further proved that nitration of caveolin-3 at amino acid residue Tyr73 leading to signal complex dissociation is indicated in the progress of cardiac insulin and adiponectin sensitivity in the prediabetic heart, thereby exacerbating the progression of ischemic heart failure [86]. Additionally, researchers revealed that hypoadiponectinemia could decrease autophagic flux and increase myocardial I/R injury under diabetes while AdipoRon, an orally molecule that binds adiponectin receptors actively [87] restores autophagosome formation via significant phosphorylation of AMPK-Beclin-1 (Ser93/Thr119)-PtdIns3K (Ser164) and partly involves with AMPK-independent signaling [88]. Similarly, researches demonstrated that a dynamic reductive AdipoR1 expression in the heart and adiponectin concentration are contributive for the increased I/R injury responsiveness while sustained insulin therapy ameliorates myocardial reperfusion injury via increasing AMPK phosphorylation in diabetic Rattus norvegicus [89, 90]. Though some researchers have put forward that adiponectin preserves the hearts from I/R injury by deterrent of inducible nitric oxide synthase (iNOS) or endothelial NO synthase (eNOS), AMPK and Akt [91, 92], The deficiency of AMPK demonstrates limited impact on the antioxidative and antinitrative protection provided by adiponectin [93]. It is well established that both hypoxia-inducible factor-1α (HIF-1α) and HO-1 serve as crucial transcriptional regulators in hypoxic cells and act as a primary role in sustaining homeostasis in cell, further investigations have found that up-regulation of HIF-1α or HO-1 could increase adiponectin expression in diabetic mouse hearts and ultimately mitigate I/R injury [94, 95]. In microRNA (miRNA) profile aspect, findings suggested that hypoadiponectinemia in diabetic Mus musculus remarkably increased miRNA-449b expression and downregulated Nrf-1 and Ucp3 levels leading to excessive ROS generation and worse myocardial I/R injury [96]. Inhibition of miR-200c-3p and activation of the AdipoR2/STAT3 signaling induced by propofol post-conditioning alleviates diabetic myocardial I/R injury [97]. Last but not the least, the ablation of CDH13 abolished adiponectin’s cardioprotective effects and increased infarct size similarly via disrupting the stimulation of a capital adiponectin signaling pathway concentrated on AMPK phosphorylation in myocardial I/R models [98]. Subsequently, the adiponectin performs its diverse functions primarily via the intricate binding mechanisms it exhibits with the AdipoR1/R2 or CDH13 receptors, and aforementioned changes of the pathophysiological mechanisms, including oxidative stress, miRNA, transcription factors, apoptosis, autophagy, and cellular signaling mentioned above, crucially regulate cardiac metabolism, affecting myocardial I/R injury under diabetes.

### Leptin

The discovery of leptin, the first adipokine synthesized and secreted by WAT encoded by the ob gene, by American scholar Jeffrey M. Friedman in 1994 marked a significant turning point in the understanding of WAT [99]. This discovery transformed the perception of WAT from a passive energy storage reservoir to a dynamic endocrine organ with active regulatory functions in behavior and metabolism. It weights 16 kDa and consists of 167 amino acids, exhibits a tertiary structure similar to that of cytokines with long chains of helices [100]. The predominant subtype of leptin receptor (LEPR), has been confirmed as the extended form that located in the hypothalamic arcuate nucleus, namely LEPRb [101]. Initially, LEPRb was deemed to be the functional receptor due to its 300 cytoplasmic residues that is longer in humans than in Mus musculus. This domain contains multiple motifs necessary for interacting with other proteins and initiating signaling pathway activation, such as the JAK-STAT3/5 and AMPK-acetyl-coenzyme A carboxylase axis [102, 103].

Study has shown that leptin acts a crucial part as the primary sensory factor for energy storage inside the human as WAT communicates with the energy metabolism to the brain through the secretion of leptin, which in turn acts on hypothalamic neurons involved in regulating appetite to suppress hunger and increase energy expenditure [104, 105]. A murine model deficient in leptin was first established in 1959, utilizing the ob/ob (leptin-deficient, caused by a single autosomal recessive mutation on the obese gene located on chromosome 6) and db/db (LEPR-deficient, caused by a single autosomal mutation on the leptin receptor gene) Mus musculus. These models have been extensively utilized in the past 20 years for the advancement of myocardial I/R injury models in T2DM [106]. Upon administering recombinant leptin to ob/ob Mus musculus, significant reductions were observed in their overall fat mass, resulting in a notable decline in food intake. Furthermore, the treatment effectively alleviated hyperglycemic and hyperinsulinemic conditions, exhibiting promising therapeutic effects in managing these metabolic abnormalities [107]. Moreover, a substantial difference is noted between Mus musculus of ob/ob and db/db genotypes and Mus musculus of wild-type genotypes in the context of post-ischemic myocardial injuries, which is attributable to multiple signal transduction pathways associated with autophagy, apoptosis, impaired insulin sensitivity, and other factors [108]. For instance, inhibition of mitsugumin 53 (MG53) E3 ligase activity mediated with MG53^S255^ phosphorylation [109], activation of PI3K/Akt pathway [110], down-regulation of histone 3 lysine 9 acetylation through histone deacetylase [111] and suppression of HMGB1-RAGE (receptor for advanced glycation end products) axis [112] are all reported to be associated with attenuation of myocardial ischemia reperfusion injury. However, leptin itself yields inconsistent consequences on myocardial I/R injury. Prior research revealed that the expression of leptin in both the serum and heart markedly decreased during the initial stage following myocardial I/R injury, followed by a gradual increase during the reperfusion phase [113]. In murine models, pre-administration with leptin resulted in a decline in cardiac and serum inflammation, improvement in myocardial reperfusion damage, and potentially involves the increase of PI3K-Akt-Nuclear Factor kappa B expression as a protective mechanism [114, 115]. During reperfusion, the manipulation of leptin caused a substantial reduction in infarction risk and a postponement in the opening of the mitochondrial permeability transition pore (MPTP), potentially mediated by the PI3K/Akt and p44/42 mitogen-activated protein kinase signaling transducer [116]. When compared to the aforementioned leptin-cardioprotective effects, respective clinical investigations have exhibited a significant correlation between diabetes and cardiovascular complications [22, 117]. To a certain extent, leptin is a inflammatory activator related to endothelial dysfunction, neointimal hyperplasia, thrombogenesis, cardiac hypertrophic and pro-remodeling [118–120]. Fortunately, later research found that leptin resistance is recognized as a remarkable risk indicator for CVD rather to leptin deficiency [20]. Excessive leptin and impaired leptin signaling shift cardiac substrate energy metabolism (glucose replaced by free fatty acid), and then trigger massive accumulation of lipid which induces lipid toxicity, poor mitochondrial functions, and increased generation of ROS in I/R injury under diabetic condition [121, 122]. Besides, insulin diminishes the storage of leptin and promotes its secretion directly in a physiological context, while leptin impedes the secretion of insulin, decreases the production and accumulation of fat, enhances the sensitivity of insulin receptors, and ultimately establishes equilibrium between fat homeostasis and energy homeostasis. Disruption of this equilibrium may lead to metabolic disturbances, insulin and leptin resistance coexist in diabetes, obesity and CVD due to both of them share the same signal transduction pathways such as protein tyrosine phosphatase 1B (PTP1B) and suppressor of cytokine signaling 3 (SOC3) [123, 124]. What’s more, researchers suggested that targeting cannabinoid (CB) receptors/modulating the degree or activity of endocannabinoids in tissue is beneficial to decrease insulin/leptin resistance in diabetes and mitigate the myocardial damage during I/R phase [125, 126]. Taken together, level of leptin could be identified as a potential diagnostic tool and label for diabetic myocardial I/R injury and managing appropriate leptin levels in individuals subjected to diabetes and myocardial I/R injury could be real fundamental for their metabolic well-being and holistic systemic health.

### Resistin

Resistin was initially identified for its role in promoting insulin resistance which controlled by the RETN gene in humans. Discovered in Mus musculus in 2001, this molecule is part of the resistin-like molecules family, characterized by a unique cysteine repeat motif (C-X11-C-X8-C-X-C-X3-C-X10-C-X-C-X-C-X9-CC-X3-6-END) and exhibiting diverse expression patterns and biological functions [127]. There is ongoing argumentation with respect to the effective impact of resistin in Mus musculus and Homo sapiens. Earlier research indicated that mouse resistin is embedded in chromosome 8 and weighing 11kDa, while Homo sapiens resistin is situated in chromosome 19 and weighing 12.5kDa, share 59% identity compared to the amino acid content, 64.4% sequence identity at the messenger RNA content, but only 46.7% sequence identity at the DNA level [128]. Moreover, resistin is commonly generated by WAT in Mus musculus, whereas in Homo sapiens, macrophages are the main origination of resistin [129].

Despite the variances between humans and rodents, there is an increasing amount of demonstrations supporting that resistin acted as a mediator in the pathogenesis of inflammatory processes and the beginning of several chronic diseases, such as metabolic malfunction, CVD, and tumor [130]. On the one hand, increased contents of resistin have been described in instances of both diet-induced obese and genetically-induced obesity while treatment of anti-resistin agent has been found to against high glucose level and to mitigate insulin resistance in experimental animals with obesity in prior studies [131]. Nagaev and his co-authors contend that there exists a dearth of correlation between insulin resistance, T2DM, and resistin expression in both adipocytes and skeletal muscle. They have noted that while resistin expression is generally low in these tissues, it can still be detected in isolated adipocytes and total WAT from certain subjects [132]. Interestingly, recent study pointed out that high content of resistin is linked to escalated mortality in T2DM and expression level of greater than or equivalent to 11ng/mL indicates an elevated risk of poor outcomes [133].

It seems that fatty acid transport protein 1, CD36, AMPK, and Acetyl-CoA carboxylase are related to insulin response and resistin function under hyperglycemia [134]. Despite that, the existing data on the effects of resistin on the myocardium have been inconclusive. Researchers demonstrated that resistin can distinctly reduce apoptotic rate and post-ischemic myocardial infarction area via PI3K/Akt/PKC or ERK1/2-matrix metalloproteinase 9 dependent pathways and thus against I/R injury [135, 136]. By contrast, studies revealed that resistin yielded non- cardioprotective effects in Langendorff-perfused rodents hearts and lacking defence in human atrial muscle subjected to reoxygenation damage [137] and resistin itself even worsens cardiac I/R injury through influencing the level of atrial natriuretic peptides during reperfusion and altering biochemical indicators of myocardial injury [138]. In addition, resistin exhibits the ability to react to two distinct receptors, toll-like receptor 4 (TLR4) and adenylyl cyclase-associated protein 1 (CAP1), thereby facilitating the initiation of inflammatory processes [139]. Currently, there is a lack of understanding regarding the potential cross-talk failure between diabetes and myocardial I/R injury. This discrepancy is connected with the significant disparities observed in the genetic and proteomic configuration of the resistin molecule between rodents and humans, and a scarcity of proof concerning the resistin receptor and its downstream signaling transducer pathways. Further assessment of the roles of resistin in patients with diabetes and myocardial I/R injury could improve the comprehension of the underlying pathways involved in the physiological and pathological development of the disease and potentially lead to improved treatment strategies for affected individuals.

### Apelin

In 1998, apelin was initially obtained from stomach extracts in bovine and characterized as a ligand for the human G protein-coupled receptor (also referred to apelin receptor (APJ)) [140]. It distributes in both human and mouse WAT, which is regulated by insulin and obesity [141]. Furthermore, apelin and APJ exhibit expression in different tissues, including but not limited to the cardiac tissue, lung, kidney, and tumor tissues, in addition to adipocytes [142]. Apelin peptides are produced by the enzymatic cleavage of a 77-amino-acid precursor molecule called pre-pro-apelin at the C-terminal end. These peptides exhibit diverse lengths in the circulating system, undergoing sequential cleavage to generate shorter, less well-defined forms, with the predominant isoforms being apelin-12, apelin-36, apelin-17, and apelin-13 [143]. Besides, these subtypes have the ability to bind APJ, however, conformational changes of receptors may impact protein function and effects [144]. And the preeminent physiologically active form of pyroglutamylated apelin-13 is the dominant apelin isoform inside the circulatory system and Homo sapiens plasma, with the capacity to potentially ameliorate vascular disease by inhibiting inflammation, suppressing apoptosis, reducing oxidative stress, and promoting autophagy [145, 146].

Apelin signaling system is tie up with a range of physiological responses, which is known to contribute to multiple pathological conditions, notably cardiovascular disorders and diabetes. In a study by Kartal and his co-workers, apelin-13 was administrated in diabetic Rattus norvegicus before blood flow caseation of the coronary artery and the erythrocyte deformability was significantly increased [147]. Moreover, they found that apelin-13 inhibits cardiac cell death and excessive inflammation activity from I/R injury in diabetic rodents [148]. A latest study showed that apelin exhibits a protective outcome against I/R injury through suppressing apoptotic rate and ROS production via the activation of PI3K and p38 mitogen-activated protein kinase signaling in diabetic hearts [149]. Given its protective activities, targeting the apelin for the treatment of CVD could be a therapeutic tool and more studies should be conducted to further explore new potential mechanisms on diabetic heart combined with I/R injury.

### Visfatin

Visfatin, (also known as NAMPT – nicotinamide phosphoribosyltransferase or pre-B cell colony-enhancing factor (PBEF)) was isolated from abdominal WAT by a Japanese research team in 2005, which is notably abundant in visceral fat tissue in Homo sapiens and Mus musculus, with its plasma expression extent rising accompanied by the progression of obesity and diabetes [150, 151]. Prior researches demonstrated that increased content of visfatin in obese and diabetic subjects may help compensate for prolonged high blood sugar by mimicking insulin and lowering glucose and lipid levels [152, 153]. A cross-sectional multicentric study revealed that visfatin in diabetic patients who received drug treatment (such as angiotensin-converting-enzyme inhibitor, calcium channel blockers or statins) couldn’t be used as a biomarker of subclinical atherosclerosis [154]. However, the function of visfatin in the physiological and pathological processes of diabetes is controversial. Prolonged visfatin treatment leading to a diabetic phenotype in Mus musculus [155] while another study reported that serum visfatin is linked to T2DM regardless of insulin resistance and obesity [156], indicating that the dual impact of visfatin on diabetes may be influenced by its concentration and requires further comprehensive clinical investigations. Furthermore, Sadoshima and colleagues conducted a series of studies examining the relation between serum levels of visfatin and I/R injury which indicated that prevention of visfatin downregulation can effectively inhibit apoptosis and stimulate autophagic flux in cardiac myocytes in response to prolonged ischemia and I/R by activating SIRT1 with upregulation of nicotinamide adenine dinucleotide (NAD^+^) and ATP contents [157–159]. Finally, Xin et al. demonstrated that treatment of visfatin reduces the inflammation and apoptosis levels of myocardial cells after myocardial I/R through activation of PI3K/AKT/ heat shock protein 70 signaling axis [160] and latest study revealed that a circular RNA associated with ferroptosis mediates the visfatin-SIRT1-FoxO1-Fth1 signaling via regulating myocardial cell ferroptosis and preserving cardiac function during reperfusion injury [161]. Li et al. suggested that activating the AMPK/NAMPT signaling improves the effectiveness of sevoflurane post-conditioning in reducing myocardial I/R injury, but this conclusion was later retracted due to inaccurate and incomplete data [162]. Off note, 1-(3,6-Dibromo-carbazol-9-yl)-3-phenylamino-propan-2-ol, known for its ability to dimerize and aggregate visfatin, has been reported to decrease the infarction area in diabetic hearts throughout cardiac ischemia and reperfusion damage recently, together with molecular signaling modification for p-AKT, phosphorylated eNOS and SIRT1 [163]. Straightforwardly, more in-depth investigations are vital to examine the latent efficacy of visfatin in diabetic myocardial I/R injury.

### Adipsin, omentin, chemerin, and metrnl

Adipokines discussed previously strongly correlate with diabetes and myocardial I/R injury. Moreover, other adipokines, including adipsin, omentin, chemerin and metrnl, have explicitly been linked to diabetes and myocardial I/R injury, respectively, providing valuable insights into the complex relationship between these two conditions. Additional research on these adipokines is necessary to support future in-depth clinical studies in this field.

Adipsin, known as complement factor D (CFD), was the first adipokine discovered by Spiegelman's research team in 1987 and subsequently determined to be a serine protease homolog produced and released by adipose cells, and is present in the circulatory system [164]. Activated adipsin has minimal proteolytic activity on most substrates, but can cleave complement factor B as it bind to energized complement factor C3, its function is homologous to that of C1s in the classical pathway [165]. In comparison to Mus musculus, human adipsin messenger RNA is also observed in monocytes and macrophages [166]. Spiegelman's lab and others later found that diabetic individuals subjected to β cell failure are lacking adipsin expression [167] and adipsin/C3a preserves β cells via lowering the phosphatase DUSP26 in diabetic Mus musculus, potentially leading to beneficial effects that are linked to a decreased risk of developing T2DM in humans [168]. Meanwhile, adipsin mitigates mitochondrial damage and enhances β-oxidation of fatty acid in diabetic cardiomyopathy through its interaction with Irak2 and impediment of Irak2 mitochondrial translocation [169]. Furthermore, exosomes originating from pericardial WAT mitigate post-myocardial infarction through adipsin-mediated regulation of iron homeostasis, while adipsin sourced from epicardial WAT contributes to cardiomyocyte apoptosis after myocardial infarction via mediation of PARP-1 activity [170, 171]. Based on the findings of a cytokine array analysis, adipsin emerges as a novel biomarker with potential utility in forecasting re-hospitalization and mortality among individuals with coronary disease [172]. Despite the promising future perspectives of the aforementioned adipsin and adipsin compounds, there are currently no robust clinical treatments available that can effectively repair myocardial reperfusion injury in diabetic individuals.

Omentin, metrnl, and chemerin are new adipokines discovered around 2005, which are mainly secreted by adipocytes to regulate the metabolism of adipocytes and exhibit either pro-inflammatory or anti-inflammatory properties in different clinical scenarios. Yang and colleagues discovered omentin (regulated by the ITLN1 gene) by analyzing 10,437 expressed sequence markers from a human omental fat cDNA library which consists of 313 amino acids and includes a secretory signal sequence as well as a fibrinogen-related domain [173]. Omentin exhibits high level in omental adipose tissue, specifically in stromal vascular cells rather than adipocytes. Its molecular mechanisms contribute to protective effects on glucose homeostasis by mitigating inflammatory processes, improving insulin sensitivity, enhancing endothelial function, and facilitating vasodilation in obesity and diabetic subjects [174]. It confers cardioprotective benefits by mitigating the progression of atherosclerosis and heart failure [175]. Furthermore, the systemic treatment of human omentin in rodents resulted in a decline in myocardial infarction risk and apoptotic rate following I/R, concomitant with increased levels of AMPK and Akt in heart [176]. The precise role of omentin remains uncertain at present; however, this molecule may serve as a noteworthy connection between diabetes and cardiovascular disease. Lastly, metformin and statins could elevate omentin-1 levels in patients [177], and thus, both of these medicines may be useful in the treatment of myocardial I/R injury under diabetic conditions.

In 2009, Surace et al. identified and documented the presence of the metrnl (311 amino acid sequence encoded by 936 base pair sequence) gene on human chromosome 17 using bioinformatics analysis [178, 179], it has become increasingly recognized as a high potential area of focus for investigation in a particular field of diabetes and CVD on recent years [180]. It is highly expressed in the skeletal muscle, subcutaneous fatty tissue, epididymal WAT depots and heart. Metrnl mitigates myocardial I/R injury-induced cardiomyocyte cell death by reducing over endoplasmic reticulum activity through the activation of AMPK-serine/threonine protein kinase pathway in cells [181]. Additionally, it improves diabetic cardiomyopathy by deactivating Cyclic GMP-AMP Synthase-Stimulator of interferon genes signaling in a manner dependent on Liver Kinase B1/AMPK/ UNC-51LikeAutophagyAckingKinase1-mediated autophagy [182]. Unfortunately, the relation between serum content of metrnl and the danger of heart disease in diabetic individuals remains inconclusive and contradictory, as evidenced by various controlled clinical trials or meta-analyses [183, 184], suggesting that metrnl content could be affected by various elements. The limited understanding of its receptor or direct interacting proteins hinders further investigation of metrnl in myocardial damage, both in the presence and absence of diabetes.

Regulated by the gene retinoic acid receptor responder protein 2 (RARRES2), chemerin is mainly generated by adipocytes to regulate the metabolism of adipocytes and exhibits proinflammatory and antiinflammatory properties through interaction with its main receptor, such as the chemokine-like receptor 1 (CMKLR1), G protein-coupled receptor 1 (GPR1) and C–C chemokine receptor-like 2 (CCRL2) [185]. It is produced in an unactive precursor form known as prochemerin, the latter is released and subjected to proteolytic cleavage by diverse extracellular proteases, resulting in the generation of distinct isoforms exhibiting varying degrees of biological activity. Down-regulation of chemerin is beneficial to reduce reperfusion injury in response to intestinal, kidney, lung and brain damage, with the primary mechanisms being associated with NLRP3 inflammasome-mediated pyroptosis [186–189]. Proof based on human and animal studies revealed that dysregulation of chemerin may serve as a risk indicator for hyperglycemia, vascular inflammation, angiogenesis, atherosclerosis, chronic heart failure and blood pressure modulation [190]. Elevated levels of chemerin have been linked to insulin resistance, disrupted blood glucose metabolism, and elevated blood glucose levels in Mus musculus [191]. Contrary to this, a recent study suggested that the addition of chemerin reversed cardiac dysfunction induced by lipid overload by increasing the messenger RNA levels of PPAR-γ and PPAR-dependent genes (such as CD36, Fabp4, and Fasn) and restoring the decrease in insulin-triggered Akt phosphorylation in Mus musculus treated with high-fat diet [192]. Additionally, a mendelian randomization study has identified potential associations between elevated genetically predicted levels of chemerin and a heightened risk of coronary disease [193]. The expanding number of research on chemerin's involvement in the pathological and physiological changes of CVD and diabetes has sparked interest in the potential use of chemerin and its associated signaling proteins as targets for the advancement of therapeutic medicines for the settlement of these conditions.

### Potential interplay among various adipokines and receptors

**Fig. 3:**
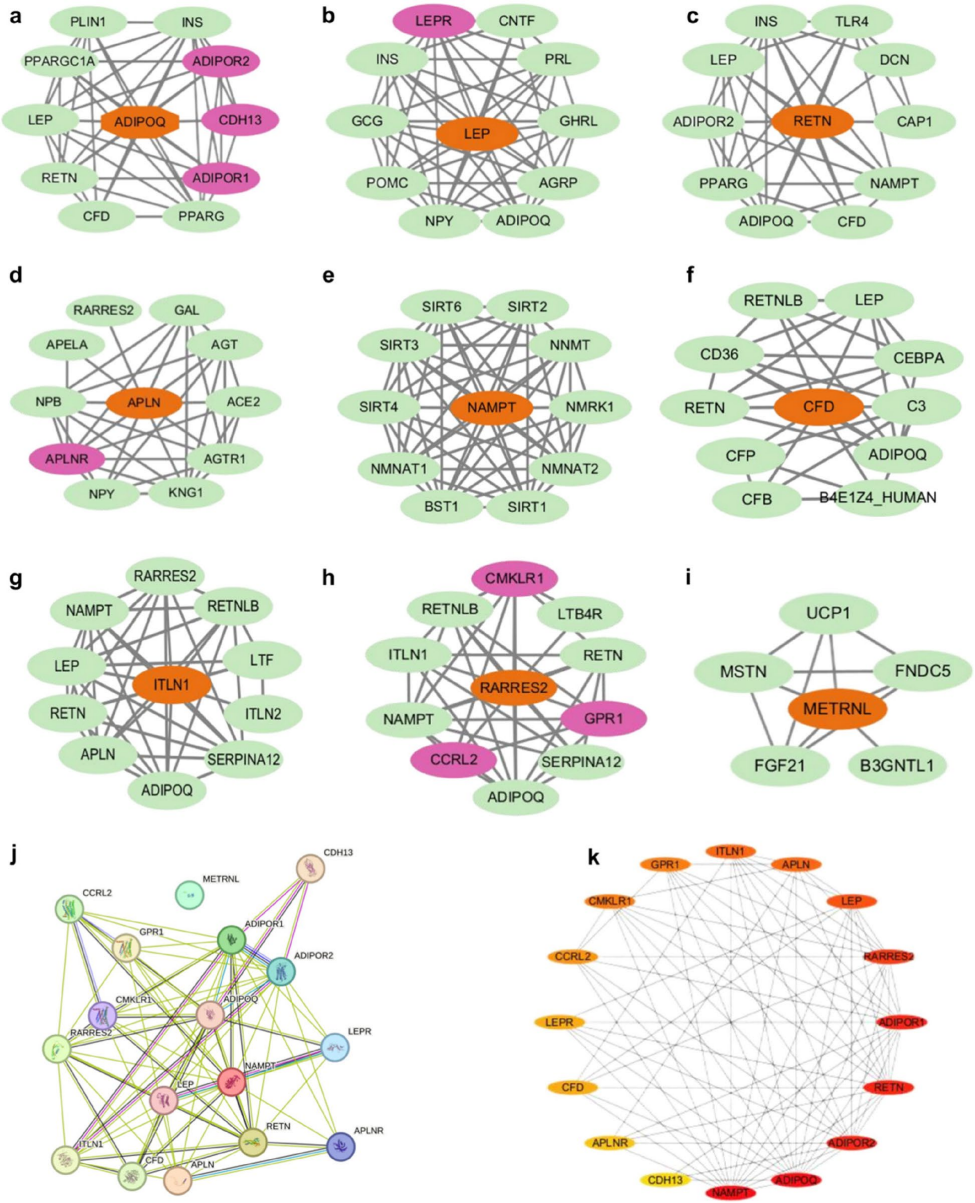
The Protein–Protein Interaction (PPI) networks of potential targets of various adipokines. a. Networks of adiponectin (ADIPOQ); b. Networks of leptin (LEP); c. Networks of resistin (RETN); d. Networks of apelin (APLN); e. Networks of visfatin (NAMPT); f. Networks of adipsin (CFD); g. Networks of omentin (ITLN1); h. Networks of chemerin (RARRES2); i. Networks of metrnl (METRNL) (a-i: the orange oval represents adipokines, the green oval denotes non-self receptor proteins, and the purple oval signifies self receptor proteins); j. Networks of various adipokines and receptors; k. Ranking of adipokines and receptors (The darker node indicates a higher ranking); (all PPI enrichment *p*-value < 0.05)

WAT is not merely a non-functional tissue but a complex and dynamic tissue that secretes adipokines in response to physiological and pathological stimuli. Due to its intricate molecular signaling pathways, WAT is crucial in maintaining body homeostasis and exerts protective or damaging effects, thus motivating and continually expanding field of research. Prior studies have primarily focused on individual adipokines or their receptors in relation to myocardial I/R injury in diabetes, rather than considering in a broader perspective. Hence, a network analysis was conducted on the aforementioned adipokines and receptors utilizing the STRING tools (http://cn.string-db.org) and Cytoscape software, aiming to outline potential interactions among adipokines in subjects who suffered myocardial I/R injury under hyperglycemia. Given the general constraint of displaying no more than a maximum of 10 interactors, it is observed that metrnl has the fewest predicted functional partners compared to the other proteins (Fig. 3a-i). Moreover, resistin exhibits the highest anticipated number of edges, with adiponectin and leptin following closely behind (Table 1). Subsequently, a more in-depth examination of the correlation between adipokines and their respective receptors was conducted, leading to the isolation of metrnl from other proteins, as the analysis was confined exclusively to the query proteins (Fig. 3j). Furthermore, a modular network was created applying the Cytohubba algorithm to reveal the core adipokine targets. The algorithm successfully identified significant network targets within the Protein–Protein Interaction (PPI) networks, with chemerin being the first target, followed by adiponectin, resistin, and visfatin.When considered collectively, adiponectin and resistin merit further investigation due to their significant potential and interconnected nature (Fig. 3k). However, this does not imply that metrnl is of lesser importance, as the PPI network is constructed using curated databases, experimentally determined data, protein homology, and other relevant factors.

**Table 1:** The results of the Protein–Protein Interaction (PPI) networks

Protein	Gene	Nodes	Edges	Average node degree	Average local clustering coefficient	Expected number of edges
Adiponectin	ADIPOQ	11	44	8	0.881	14
Leptin	LEP	11	50	0.09	0.923	14
Resistin	RETN	11	39	7.09	0.862	15
Apelin	APLN	11	36	6.55	0.907	12
Visfatin	NAMPT	11	52	9.45	0.958	11
Adipsin	CFD	11	33	6	0.859	12
Omentin	ITLN1	11	41	7.45	0.872	11
Chemerin	RARRES2	11	37	6.73	0.796	11
Meteorin-like protein	METRNL	6	11	3.67	0.933	5

## Limitation and considerations

Based on empirical evidence, among the adipokines discovered thus far, adiponectin, leptin, resistin, and apelin emerge as the most promising candidates for clinical application, particularly in the realms of myocardial protection and diabetes management. Yet, numerous potential avenues for further investigation persist, as the existing data exhibit several inconsistencies.

Elevated levels of adiponectin, known for its against inflammatory and cardioprotective effects, are linked to a declined risk of cardio damage in individuals with diabetes. Animal studies provide evidence supporting adiponectin as a cardioprotective protein in cardiovascular health [194], on the contrary, a two-year investigation found that heightened serum concentrations of adiponectin were linked to an increased likelihood of cardiovascular events leading to elevated mortality rates [195]. Besides, high-molecular-weight adiponectin is concerned with higher mortality in elder subjects compared with healthy middle-aged populations [196]. Furthermore, the expressed level of adiponectin receptors in human are influenced by gender, with males exhibiting significantly higher levels compared to females. Conversely, females demonstrate higher serum adiponectin levels than males [197]. Despite these conflicting associations, commonly referred to as the "adiponectin paradox" and previously elucidated factors such as renal dysfunction, adiponectin resistance, weight change, and hydrolysis of CDH13 [198, 199], adiponectin still remain a major puzzle in the field. Consequently, it is imperative to deepen the understanding of adiponectin's precise function and assess its potential to strike a balanced approach that minimizes diabetic myocardial I/R injury and mortality by explicitly targeting the cardiac adiponectin signaling pathway. Unlike other adipokines, adiponectin in blood does not seem to be affected by atorvastatin treatment in patients [200]. Lastly, adiponectin levels were independently associated with restenosis, but both HOMA-IR (Homeostatic model assessment of insulin resistance) and adiponectin were independently associated with de novo ischemic heart disease and the incidence of new percutaneous coronary interventions in patients with normal glucose tolerance [201].

The ob/ob and db/db rodent genotypes serve as representations of diabetes as a monogenic disorder, contrasting with the polygenic and multifactorial nature of human T2DM [202]. For instance, hyperglycemia progresses gradually and deteriorates over time in humans, while blood glucose levels exhibit transient and limited severity in ob/ob Mus musculus, with not all db/db Mus musculus experiencing the development of hyperglycemia [203, 204]. Consequently, although these models are valuable for investigation purposes, the findings may have limited applicability, especially in diabetic myocardial I/R injury. Leptin’s effects are currently incongruous and lacks comprehensive understanding, it is widely agreed upon that both elevated expression of leptin and leptin deficiency may have potential impacts for CVD. In-depth research endeavors are imperative in order to definitively ascertain whether these effects are being fine-tuned by distinct molecular signaling pathways or particular receptor isoforms. Certain hypoglycemic agents, including metformin and sodium-glucose cotransporter 2 (SGLT2) inhibitors, have been documented to enhance cardiac outcomes. Research indicates that metformin therapy, by reducing pericoronary fat levels, contributes to improved cardiovascular outcomes through the diminution of inflammatory markers, SGLT2, and leptin levels in individuals with pre-diabetes [205]. Furthermore, another study suggests that SGLT2 inhibitors may mitigate the inflammatory profile in patients with diabetes [206]. Take together, there appears to be a potential association between leptin and SGLT2.

Due to the insufficient availability of reliable and comprehensive data on resistin, it cannot be considered as a reliable independent predictor of either diabetes or CVD. However, a clinical trial showed that levels of several adipokines significantly changed in individuals suffering coronary artery bypass graft surgery with cardiopulmonary bypass, with concentrations of adiponectin and adipsin diminished, but levels of leptin and resistin significantly augmented within 24 h following the commencement of the operation [207]. Combination of several kinds of adipokines may act as a functional biomarker or risk predictor in I/R injury. Adiponectin-resistin (AR) index (fasting serum total adiponectin and resistin levels) and insulin resistance-AR (IRAR) index (integration of the AR index into an existing insulin resistance index) have been used to screen individuals with elevated risk of potential progress of T2DM and metabolic syndrome before [208], recent study found that both of them applies on cardiovascular risk in diabetic patients as well [209]. The indices pertaining to AR and IRAR underwent a marked and significant upsurge in diabetic group compared with control group and further analysis demonstrated that these indices calculated level of cardiovascular risk through area under the curve [101]. Besides, adipose-derived stem cells (ADSCs) are regarded as potential instruments for the replacement, repair, and regeneration of necrotic or impaired cells [210]. He et al. utilized ADSCs in a murine model of I/R injury, employing both resistin-treated and vehicle-treated ADSCs. Their findings indicated that ADSCs treated with resistin significantly enhanced myocardial ejection fraction and reduced myocyte apoptosis [134]. ADSCs have been the subject of numerous Phase I and II clinical trials, including the use of a transendocardial delivery system for administering stromal vascular fraction to the akinetic myocardial scar region [211], as well as intra-articular injections of allogeneic ADSCs for the treatment of knee osteoarthritis [212]. Nonetheless, the intravenous administration of ADSCs has demonstrated limited retention and survival rates of myocardial stem cells [213].

Apelin has been suggested as a novel biomarker for prognostication in myocardial ischemic patients with ST-segment elevation, with studies indicating that elevated plasma levels of apelin upon admission are in connection to a significant risk of mortality at the 6-month follow-up, thus augmenting the prognostic value provided by brain natriuretic peptide [214]. Certain researchers have posited that the endogenous release of the peptide may serve to mitigate the extent of an infarction [215] and the apelin/APJ system functions to mitigate imbalanced oxidative reaction between oxygen and lipid in mitochondrial by facilitating the formation of nitric oxide during myocardial reperfusion damage as well [216]. Furthermore, the clinical utility of apelin as a therapeutic agent is restricted by its brief half-life and the requirement for parenteral delivery. Various studies have been undertaken to investigate potential small molecule apelin agonists, yet only a few have been progressed to further evaluation [217]. Given that Apelin has been shown to interplay with caveolin in cardiomyocytes [218], it is possible that apelin may interact with adiponectin via caveolin in the context of myocardial I/R injury in diabetes, although further study is needed to test this hypothesis.

## Conclusion

Taken together, adipokines should be regarded as prospective therapeutic targets for CVD, necessitating further research on optimizing adipokine levels to mitigate the systemic impact of adipokines on myocardial I/R injury in subjects with diabetes. It is advisable to begin monitoring the dynamic changes of blood adipokines in diabetic patients, given that the current investigation of various adipokines lacks a comprehensive analysis of preclinical and clinical data. Meanwhile, much deeper research is necessary to investigate potential molecular mechanisms underlying the co-occurrence of diabetes and myocardial I/R injury, mainly focusing on the interaction between oxidative response, lipid imbalance, and programmed cell death pathways. Developing small-molecule adipokine compounds, including agonists and inhibitors or synthetic adipokine analogs, is recommended to facilitate future clinical studies in this area.

## Supplementary Information

Supplementary Material 1.

Supplementary Material 2.

## Data Availability

No datasets were generated or analysed during the current study.

## Abbreviations

CVD: Cardiovascular disease
I/R: Ischemia/reperfusion
WAT: White adipose tissue
Metrnl: Meteorin-like
JAK: Janus-activated kinase
STAT3: Signal transducers and activators of transcription 3
AMPK: AMP-activated protein kinase
ROS: Reactive oxygen species
RISK: Reperfusion Injury Signaling Kinase
PI3K: Phosphoinositide 3-kinase
Akt: Protein kinase B
ERK1: Extracellular signal-regulated kinase 1
ERK2: Extracellular signal-regulated kinase 2
MAPK: Mitogen-activated protein kinases
SAFE: Survivor Activating Factor Enhancement
PTEN: Phosphatase and Tensin Homolog
JAK2: Janus-activated kinase 2
FoxO: The class O of Forkhead box
CD36: Cluster of differentiation 36
PKC: Protein kinase C
HMGB1: High mobility group box1protein
SIRT1: Sirtuin 1
Nrf2: Nuclear factor E2-related factor 2
HO-1: Heme oxygenase-1
PPAR: Peroxisome proliferator-activated receptor
p-Akt: Phosphorylated protein kinase B
Enos: Endothelial nitric oxide synthase
T1DM: Type 1 diabetes mellitus
T2DM: Type 2 diabetes mellitus
MG53: Mitsugumin 53
mPTP: Mitochondrial permeability transition pore
AdipoR1: Adiponectin receptor 1
AdipoR2: Adiponectin receptor 1
CDH13: T-cadherin
PPAR: Peroxisome proliferator-activated receptor
p-Akt: Phosphorylated protein kinase B
iNOS: Inducible nitric oxide synthase
HIF-1α: Hypoxia-inducible factor-1α
miRNA: MicroRNA
LEPR: Leptin receptor
PTP1B: Protein tyrosine phosphatase 1B
SOC3: Suppressor of cytokine signaling 3
CB: Cannabinoid
TLR4: Toll-like receptor 4
CAP1: Adenylyl cyclase-associated protein 1
APJ: Apelin receptor
PBEF: Pre-B cell colony-enhancing factor
NAD^+^: Nicotinamide adenine dinucleotide
CFD: Complement factor D
RARRES2: Retinoic acid receptor responder protein 2
CMKLR1: Chemokine-like receptor 1
GPR1: G protein-coupled receptor 1
CCRL2: C chemokine receptor-like 2
PPI: Protein-Protein Interaction
AR: Adiponectin-resistin
IRAR: Insulin resistance-AR
SGLT2: Sodium-glucose cotransporter 2
ADSCs: Adipose-derived stem cells
