# Supplementary material for: Adipokines and their potential impacts on susceptibility to myocardial ischemia/reperfusion injury in diabetes
Source: Lipids Health Dis. 2024 Nov 13;23:372. doi: 10.1186/s12944-024-02357-w (PMC11558907; doi:10.1186/s12944-024-02357-w)
Supplement: Supplementary file 1 — Supplementary Material 1. [file 12944_2024_2357_MOESM1_ESM.pdf]

Adipokines and their potential impacts on susce...

By: Ronghui ronghui

As of: Oct 29, 2024 6:26:29 PM
8,686 words - 138 matches - 119 sources

Similarity Index

14%

Mode: Summary Report

paper text:

Abstract Coronary artery disease has a high mortality rate and

is a striking **public health concern** , affecting **a substantial** portion of **the** 26

global population. On the early onset of myocardial ischemia, thrombolytic therapy and coronary revascularization could promptly restore the bloodstream and nutrient supply to the ischemic tissue, efficiently preserving less severely injured myocardium. However, the abrupt re-establishment of blood flow triggers the significant discharge of previously accumulated oxidative substances and inflammatory cytokines, leading to further harm referred

to as ischemia/reperfusion (I/R) injury. Diabetes significantly raises **the** vulnerability **of** 61

the heart to I/R injury due to disrupted glucose and lipid processing, impaired insulin sensitivity and metabolic signaling, and increased inflammatory responses. Numerous studies have indicated that adipokines are crucial in the etiology and pathogenesis of obesity, diabetes, hyperlipidemia, hypertension, and coronary artery disease. Adipokines such as adiponectin, adipsin, visfatin, chemerin, omentin, and apelin, which possess protective properties against inflammatory activity and insulin resistance, have been shown to confer myocardial protection in conditions such as atherosclerosis, myocardial hypertrophy,

myocardial I/R injury , and diabetic complications. **On the other hand** 1

, adipokines such as leptin and resistin, known for their pro-inflammatory characteristics, have been linked to elevated cardiac lipid deposition, insulin resistance, and fibrosis. Meteorin-like (metrnl) exhibits opposite effects in various pathological conditions. However, the data on adipokines in myocardial I/R, especially in diabetes, is still incomplete and controversial.

This review focuses on recent research regarding **the** categorization **and function of** adipokines **in** the heart muscle, **and** 26

the identification of different signaling pathways involved in

**myocardial I/R injury** under **diabetic conditions** , aiming **to** facilitate **the** exploration **of**	1

therapeutic strategies against 1

**myocardial I/R injury in** diabetes. **Keywords: diabetes, myocardial ischemia/reperfusion (I/R) injury**	51

, adipokines Introduction As claimed by the most recent American Heart Association report, mortality rates stemming from cardiac and cardiovascular disease (CVD) have declined by 60% since 1950 as a result of precise diagnostic techniques and proactive medical and surgical interventions in the United States [1]. Coronary artery bypass grafting off or on-pump and direct percutaneous coronary intervention are widely recognized as the most efficacious therapeutic modalities for myocardial preservation in instances of cardiac injury [2,3]. It is essential to understand that the sudden reintroduction of oxygen and nutrients can disturb the function and electrical activity of the heart muscle, leading to damage and exacerbating myocardial necrosis, a condition referred to as

**myocardial ischemia/reperfusion (I/R) injury [4]. In** light **of**	45

recent advancements in research on the molecular mechanisms

**of myocardial I/R injury** , significant attention **has been** paid **to** elucidating the role **of**	1

mitochondrial, lipid and glucose metabolism, oxidative products, calcium regulation, and cell signaling [5 , 6]. However, there remains a lack of effective treatments for this condition in medical settings. Conversely, there has been a slow increase in mortality rates from the end of the 2010s to 2020, which is

**due to the** worsening **of risk** hazards **such as diabetes** , hypertension, **obesity**	71

, aging population, and other related factors [1]. What is more, the global prevalence of diabetes reached 529 million individuals in 2021, with projections indicating a significant increase to 1.31 billion by the year 2050 [7]. Recent research indicates that diabetes can heighten the heart's responsiveness to I/R damage, reduce

the heart's ability to respond to protective **measures** 94

, disrupt energy metabolism, worsen the oxidative response and inflammatory activity in the heart, and consequently raise 2 the likelihood of cardiomyocyte death through various mechanisms like apoptosis, necroptosis, ferroptosis, and pyroptosis [8,9]. It is crucial to comprehend the pathogenic process that controls the advancement and worsening of myocardial I/R injury under hyperglycemia and to investigate reliable cardiac biomarkers for predicting risk. Previously considered solely as a non-active energy receiver,

white adipose tissue (WAT) has recently **been** acknowledged **as** an essential **endocrine** 81 component **that**

generates multiple peptide hormones with autocrine, paracrine, or endocrine effects on diverse physiological processes [10,11]. These secretions comprise a varied assortment of small chemical molecules, such as cytokines and chemokines, that engage with adipose cells, immune cells, and non-regenerative cells (osteoblasts, neurocytes, retinal cells, pancreatic β cells, and cardiomyocytes) [12]. Certain agents are generated by cells other than adipocytes. In contrast, others are secreted by adipocytes and categorized as adipokines that include but are not limited to adiponectin, leptin, resistin, apelin, adipsin, visfatin, omentin, chemerin and meteorin-like (metrnl) [13]. Recent research has demonstrated that adipokines

have a multifaceted **impact on** the **insulin sensitivity** , atherosclerosis, **inflammation,** 113 **and**

myocardial signaling pathogenesis. They display seemingly contradictory effects on the heart's functionality, particularly after oxidative products and I/R injury [14,15,16]. In a comprehensive analysis of preclinical animal studies, it was observed that adiponectin, possessing insulin-sensitizing and anti- inflammatory attributes, effectively suppressed apoptosis in cardiac muscle cells exposed to reperfusion injury by stimulating diverse molecular pathway cascades [17]. For instance, a study proposed that administering adiponectin as a supplement could potentially strengthen the responsiveness

of the diabetic heart to ischemic post- conditioning through **the** initiation **of** 47

diverse cellular

signaling pathways, including **Janus-activated kinase** (JAK) / **signal transducers and activators of transcription** 3 3 (STAT3) **and** AMP- **activated protein kinase** 48

(AMPK) [18]. Besides, intracerebroventricular administration of leptin significantly mitigated cardiac malfunction following I/R injury, as proved by enhanced ventricular systolic function, overall cardiac function, and mitochondrial metabolism [19]. Therefore, evidence suggests that adipokines are vital in developing myocardial I/R injury in subjects with or without diabetes, but its molecular mechanism remains largely unclear. Previous research has predominantly concentrated on individual adipokines or their receptors

in relation to myocardial I/R injury or diabetes, highlighting **the** significant impact **of** 75

adipokines on cardiovascular disease through glucose and lipid metabolism disorders. By contrast, this review mainly examined the regulation and effects of different adipokines and associated signaling pathways and discussed their potential impacts on myocardial I/R injuries. Furthermore, we also performed a network analysis of the different adipokines and the relevant receptors utilizing the STRING tools and Cytoscape software, aiming to outline potential interactions among adipokines

in the context of myocardial I/R injury in diabetic individuals **and** conducted comprehensive research **to** facilitate **the** development **of** 53

innovative therapeutics and preventive measures. 1.

Increased vulnerability to **myocardial I/R injury in diabetes** 17

Diabetes, a metabolic disorder, is marked **by high blood sugar levels** resulting from insufficient **insulin** 62

secretion or activity. It affects a substantial portion of the worldwide populace and is associated with various complications, particularly cardiovascular disease [20]. Of these complications, myocardial I/R injury is a prominent issue. Tissue damage arises as a consequence of the bloodstream supply briefly halted and then restored. Diabetic individuals are more sensitive to

myocardial I/R injury, and the mechanisms driving this **are** discussed **in the** 63

following sections. 4 1.1 Clinical perspective Numerous studies have consistently shown that individuals with diabetes exhibit a heightened sensitivity to myocardial I/R injury when compared to non-diabetic counterparts [21 , 22]. This increased risk is attributed to various underlying pathophysiological mechanisms, including hyperglycemia/hyperlipidemia, insulin resistance, compromised coronary microcirculation, endothelial dysfunction, elevated oxidative products, and dysregulated inflammatory activities [23]. Hyperglycemia

is associated with an imbalanced **generation of reactive oxygen species** (ROS) **and** 79

antioxidant defense malfunction, resulting in oxidative stress and exacerbating myocardial damage during periods of ischemia and reperfusion [24]. Studies from our laboratory illustrated that high glucose increased myocardial infarct area during I/R in Mus musculus and enhanced ROS generation in vivo and in vitro [25,26]. Insulin resistance, a prevalent characteristic of diabetes, hinders the uptake and utilization of glucose in cardiomyocytes, resulting in disrupted energy metabolism, mitochondrial dysfunction, and heightened susceptibility to myocardial injury [27]. Meanwhile, insulin resistance exacerbates inflammation and endothelial dysfunction, exacerbating myocardial dysfunction and augmenting susceptibility to I/R injury [28]. Diabetes- related impairment of angiogenesis impedes the development of new blood vessels in the ischemic myocardium, leading to reduced oxygen and nutrient delivery to the affected region, thereby exacerbating myocardial injury during reperfusion [29]. Additionally, endothelial dysfunction and microvascular abnormalities induced by diabetes contribute to impaired coronary flow reserve, thereby restricting the myocardium's capacity to withstand ischemic insults [30,31]. There is compelling evidence indicating that healthy platelets possess cardioprotective properties; however, studies have shown that platelets obtained from sufferers with poorly managed type 2 5 diabetes mellitus (T2DM) exhibit diminished beneficial properties compared to platelets from healthy individuals [32]. Activated platelets migrate into the damaged cardiac muscle and provoke I/R injury by forming micro-emboli-small clots, enhancing platelet-leukocyte aggregation, and releasing vasoconstrictor and pro-inflammatory mediators [33]. Lastly, diabetic patients frequently present with comorbid conditions such as hypertension and dyslipidemia, which further increase their vulnerability to myocardial I/R injury [34]. Effective management of diabetes and its related cardiovascular complications is essential in minimizing the risk of myocardial I/R injury. Maintaining tight control over blood sugar levels (lifestyle changes, oral hypoglycemic medications, or insulin therapy) has been demonstrated to decrease ROS generation, inflammation, and endothelial dysfunction, ultimately reducing the area of cardiac damage during I/R [35]. Nevertheless, it is essential to acknowledge that strict glucose control may have constraints, as evidenced by previous studies demonstrating an elevated risk of cardiac attack in patients undergoing specific anti-hyperglycemic drug therapies [36], and severe hyperglycemia was more common in the strict-glucose-control group [37]. One possible explanation for this phenomenon might be the crucial character of myocardial glucose uptake and metabolism in sustaining myocardial energetic during periods of stress [38,39].

1.2 Underlying **molecular mechanisms of** increased vulnerability to **myocardial I/R injury** in 1
diabetes **The** increased vulnerability to **myocardial I/R injury**

in diabetes to **myocardial I/R injury in** individuals **with diabetes is** 64

a multifaceted phenomenon that is impacted by a variety of pathophysiological mechanisms. In other words, it is imperative to comprehend these underlying molecular mechanisms in order to formulate precise therapeutic approaches

to mitigate myocardial damage and to enhance **clinical** 6 **outcomes in** 88

diabetic populations. The fundamental mechanisms of diabetes aggravating myocardial I/R injury are summarized in the Fig. 1. Research indicates that the diabetic heart may resist cardioprotective interventions. This could be attributed to disruptions in various signaling pathways and abnormal cardiomyocyte death, as observed in animal studies and in in vitro experiments, although clinical confirmation is currently insufficient [40]. The Reperfusion Injury Signaling Kinase (

RISK) pathway, which encompasses the phosphoinositide 3-kinase (PI3K) /protein kinase B (5
Akt) and extracellular signal-regulated **kinase** 1 (ERK1) / **extracellular signal-regulated**
kinase

2 (ERK2), mitogen-activated protein kinases (**MAPK) signaling pathway, and** 40

the Survivor Activating **Factor Enhancement (SAFE) pathway, which** includes **the** 56
JAK/STAT3 signaling cascade

, play crucial roles in myocardial protection [41]. Impairments in above-mentioned signaling transducers diminish the responsiveness of diabetic myocardium to therapeutic interventions. Many studies have explored the cardiac protective effect of

RISK and SAFE during the reperfusion period **after the** 101

lethal ischemic insult. In this regard, our group's findings indicated that activation of these two pathways following the onset of diabetes effectively reduced the size of infarct area caused by I/R and cell death triggered by high glucose and hypoxia/reoxygenation [42]. We further revealed that targeted inhibition

of Phosphatase and Tensin Homolog (PTEN) with the 30

inhibitor bisperoxovanadium maintained the cardioprotective efficacy of post-ischemic- conditioning in streptozotocin-induced diabetic Rattus norvegicus by restoring the

PI3K/Akt and Janus -activated **kinase 2 (JAK2)/STAT3 pathways** 32

[43], suggesting that the heart may regulate the phosphorylation of these kinases through the activation of PTEN under diabetic conditions. However, whether these two pathways may interact or be totally independent during myocardial I/R remains unclear. It appears that STAT5, 7 as opposed to STAT3, may be a significant factor in the process of cardioprotection in the context of human physiology [44,45]. Additionally, both

the class O of Forkhead box (FoxO) transcription factors and 60

the cluster of differentiation 36 (CD36)/AMPK signaling are involved in interventions combating against myocardial I/R injury via inhibiting excessive apoptotic cells, autophagy and ferroptosis as have been described in our recent studies [25,26]. Recently, ferroptosis has emerged as an unique

form of iron- dependent necrosis that differs **from apoptosis, autophagy, and other** established 9
mechanisms **of cell death**

. To date, several studies have validated the presence of myocardial cell ferroptosis in diabetic animal models, evidenced by the facts that administration of the ferroptosis agonist Erastin exacerbated myocardial I/R injury and post-ischemic cell death while

the ferroptosis inhibitor Ferrostatin-1 or **the** utilization **of the** 102

antioxidant N-acetylcysteine

has been shown to attenuate myocardial I/R injury 23

under high glucose condition [46,47,48]. AMPK, protein kinase C (PKC), ERK1/2, PI3K, and Akt defend against ferroptosis in myocardial tissue [49], while phosphoenolpyruvate carboxykinase-α/β (PCKα/β) inhibition also yield cardioprotective effects under high glucose [50]. In addition to the signaling mentioned above pathways and programmed cell death mechanisms, a variety of intracellular signaling molecules and cell death processes can also trigger myocardial I/R injury when hyperglycemia is present, such as necroptosis induced by

necrosis-related proteins (Caspase 3, Bax, p-RIP3, p-RIP1, and p-MLKL) through **the** 35

JAK2/STAT3 pathway [51], pyroptosis induced by the NLRP3 inflammasome via AMPK- Nicotinamide adenine dinucleotide phosphate (NADPH) oxidase-2 signaling [52], autophagy regulated by high mobility group box1protein (HMGB1) [53],

sirtuin 1 (SIRT1) /nuclear factor E2-related factor 2 (Nrf2)/heme oxygenase-1 (HO-1 8

) pathway activated by exaggerated generation of ROS and poor mitochondrial function [54], 8 endothelial and vascular dysfunction with the suppression

of endothelial nitric oxide synthase (eNOS) [55], inhibition of **ROS-induced** 99

apoptotic rate by activating

the AMPK/Akt/GSK-3β (glycogen synthase kinase-3β signaling 67

) and Nrf2-governed antioxidant enzymes activity [56].

Fig. 1. Mechanism of diabetes mellitus aggravating myocardial ischemia/reperfusion (I/R) **injury** 43

. Diabetes mellitus is categorized predominantly into

type 1 diabetes mellitus (T1DM), distinguished by insufficient **insulin** production, and **type 2 diabetes mellitus (T2DM** 10

), distinguished by reduced sensitivity to insulin. Diabetic condition primarily leads to hyperglycemia, hyperlipidemia, disrupted energy metabolism, endothelial dysfunction, and a range of associated complications. Under situation of myocardial ischemia, decreased ATP levels, increased

opening of the mitochondrial permeability transition pore (mPTP), and **the** subsequent burst **of** ROS during **myocardial reperfusion** 57

contribute to diverse types of cell deaths (including pyroptosis, necrosis, autophagy, ferroptosis, and apoptosis). Diabetes exacerbates myocardial I/R injury primarily through mechanisms involving inflammatory response, oxidative stress, disrupted mitochondrial and microvascular function and the key signal transduction pathways indicated in this action are the RISK and SAFE pathways. Current research indicates that the predominant pro-survival signaling involved are FoxO, AMPK, SIRT1, Nrf2, HO-1, GSK-3β and eNOS and the anti-survival signaling are primarily PTEN, CD36, HMGB1 and Nox2, while PKC exhibits dual function. FoxO: the class O of Forkhead box

1; AMPK: AMP-activated protein kinase; SIRT1:sirtuin 1 31

;

Nrf2: nuclear factor E2- **related factor 2; HO-1: heme oxygenase-1; GSK** -3β: **glycogen synthase kinase** -3β; **eNOS: endothelial nitric oxide synthase** 42

; PTEN: Phosphatase and Tensin Homolog; CD36:cluster of differentiation 36; HMGB1: high mobility group box1protein; Nox2:

Nicotinamide adenine dinucleotide phosphate (NADPH) oxidase -2 ;PKC: **protein kinase C** 80

;

ERK 1: extracellular signal-regulated kinase 1; ERK2: extracellular signal-regulated kinase 2 25

; JAK: janus-activated kinase;

STAT3: signal transducers **and** activators **of transcription 3; PI3K:phosphoinositide 3-kinase; Akt: protein kinase B** ; MAPK: mitogen-activated **protein** 39

kinase. 2. Impacts of adipokines

on diabetes complicated **with myocardial I/R injury** Various hormone- **like** 43

molecules (

adiponectin, leptin, resistin , apelin, **visfatin** , adipsin, **omentin, chemerin, and** metrnl) **are** 105

classified as adipokines that are produced by WAT and

play a multifaceted **role in** numerous **diseases, including** diabetes, **atherosclerosis** , CVD, **and** 47

immune disorders [57,58]. In obese pre-diabetic patients, abdominal fat tissue

is a significant **source of inflammatory and oxidative stress metabolites** 70

. Previous study found that high levels of these molecules are linked to low SIRT1 expression in adipose tissue, potentially impacting heart function both locally and systemically [59]. Furthermore, epicardial fatty tissue has been pinpointed as a leading source of CVD and metabolic disorders after an acute coronary event and coronary inflammation in T2DM given that it is closely adjacent to the coronary arteries [60,61]. Besides,

women have unique atypical risk factors that are associated with the prognosis of CVDs, such as pre-menopausal breast fat accumulation 16

[62]. Adipokines have been implicated as playing both protective and deleterious effects of I/R injury by influencing various molecular pathways (Fig. 2). Myocardial I/R injury and diabetes are both associated with several mechanistic

aspects of adipokines that have yet to be clarified. Fig. 2. The function and main molecular mechanism of various adipokines on myocardial ischemia/reperfusion (I/R) injury. Adipokines are mainly divided into two categories: anti-inflammatory (including adiponectin, adipsin, visfatin, chemerin, omentin and apelin, related signaling pathways are marked in each corresponding yellow box) and pro-inflammatory (leptin and resistin, related signaling pathways are marked in each corresponding blue box), while metrnl (related signaling pathway are marked in red box) exhibit opposite effects in various pathological condition. WAT: white adipose tissue;

**JAK2: janus** -activated **kinase 2; STAT3: signal** transducers **and** activators **of transcription 3; PI3K:phosphoinositide 3-kinase; Akt: protein kinase B**	3

;

**AMPK: AMP-activated protein kinase; PPAR: peroxisome proliferator-activated receptor**	38

;

**HIF-1α: hypoxia-inducible factor-1α; HO-1: heme oxygenase-1**	59

; miRNA: microRNA; NO synthase:

**nitric oxide synthase; HSP70: heat shock protein 70** ; SIRT1: sirtuin **1**	85

; FoxO1: the class O of Forkhead box 1; NAD+ : nicotinamide adenine dinucleotide; p-eNOS:

**phosphorylation of endothelial nitric oxide synthase** ; p- **Akt** : phosphorylation **of**	7

**protein kinase B; p38/MAPK: p38 mitogen-activated protein kinase**	22

; CD36: cluster of differentiation 36; PAK2: serine/threonine protein kinase

**2; cGAS-STING: Cyclic GMP-AMP Synthase-Stimulator of interferon genes**	36

; LKB1: Liver Kinase B1; ULK1: UNC-51LikeAutophagyAckingKinase1; PKC: protein kinase C; ERK 1:

**extracellular signal-regulated kinase 1 ; ERK2: extracellular signal- regulated kinase 2; MMP** -9: **matrix metalloproteinase**	21

9;

**TLR4: toll-like receptor 4; CAP1: adenylyl cyclase-associated protein 1**	44

; MG53: mitsugumin 53; HMGB1: high mobility group box1protein;

**NFκB: Nuclear Factor kappa B; MAPK: mitogen- activated**	117

**protein kinase; PTP1B: protein tyrosine phosphatase 1B** ; SOC3: **suppressor of cytokine** signaling **3**	58

;

**CB1: Cannabinoid Receptor 1; CB2: Cannabinoid Receptor 2. 2**	82

.1 Adiponectin An independent research study employing various experimental methodologies initially characterized adiponectin, a protein uniquely generated by adipose cell and regulated by the ADIPOQ gene, known by various aliases including GBP-28, AdipoM1, AdipoQ, and Acrp30 in humans [63,64,65,66]. It typically exhibits high levels in the bloodstream, ranging from

**3-30μg/mL, and** comprises only **0.01% of** pure protein in **plasma**	98

, in comparison to else hormones and cytokines [67]. Meanwhile, adiponectin contains 247 amino acids including signal sequence, unstable domain, collagen-like domain, globular domain and it subsists in trimers (the fundamental unit), consists of hexamers with low molecular weights and isoforms with high molecular weights [68]. Until now, there are three central

adiponectin receptors, including adiponectin receptor 1 (AdipoR1), adiponectin receptor 2 (AdipoR2) and T-cadherin (CDH13 37

). AdipoR1 demonstrates a strong binding affinity towards globular adiponectin, whereas its interaction with full-length adiponectin is relatively weak. On the other hand, adiponectin with globular or full-length exhibits a moderate adaptability for AdipoR2 [69]. In terms of CDH13, it

has been regarded **as a receptor for hexameric and high molecular** weights **of** isoforms of **adiponectin** 52

[70]. Plenty of clinical trials have implied that individuals with elevated adiponectin levels have less chance of diabetes and are less susceptible to CVD [71,72]. Meanwhile, animal experiments have indicated that insulin resistance is improved noticeably and post-ischemic myocardial infarction reduced significantly with exogenous adiponectin or adiponectin receptors agonist [73,74]. For instance, previous studies have reported that cardiomyocytes from adiponectin knockout Mus musculus sustained severer I/R injury which could not be reverted or ameliorated by

peroxisome proliferator-activated receptor (PPAR)-γ or **PPAR-γ** 24

agonist [75,76,77]. Furthermore, cardiac adiponectin could act as an adjuster via AdipoR2 to prevent diabetic myocardial I/

R injury through PI3K/Akt and JAK2/STAT3 116

[42,78]. Furthermore, Cao et al illustrated that ischemic postconditioning contributed to a significant loss in post-ischemic myocardial infarction and ROS accumulation in normal Rattus norvegicus, a phenomenon closely linked to increased expression of adiponectin

and phosphorylated protein kinase B (p- Akt). The 91

above conducive outcomes were canceled in diabetic Rattus norvegicus and the expressions of adiponectin were restrained [79]. Moreover, Li et al proposed that the protective consequences of ischemic postconditioning are compromised in diabetes as a result of impaired adiponectin/AdipoR1/caveolin-3 signaling [80]. It is noted that 13 caveolin has been generally identified as a tremendous latent spot in multifarious biological processes, including adiponectin signalosome formation and cardiac protection [81]. Wang et al. have shown that the knockout of caveolin-3

significantly blunts adiponectin's anti-apoptotic effect and exacerbates myocardial I/R injury. AdipoR1 co-localizes with caveolin-3 to form a complex, and the latter activates adiponectin cardioprotection signaling pathways, which are regulated by AMPK or not [82]. Recently, a study further proved that nitration of caveolin-3 at amino acid residue Tyr73 leading to signal complex dissociation is indicated in the progress of cardiac insulin and adiponectin sensitivity in the prediabetic heart, thereby exacerbating the progression of ischemic heart failure [83]. Additionally, researchers revealed that hypoadiponectinemia could decrease autophagic flux and increase myocardial I/R injury under diabetes while AdipoRon, an orally molecule that binds adiponectin receptors actively [84] restores autophagosome formation via

**significant phosphorylation of AMPK** -Beclin-1 (**Ser93/Thr119)-PtdIns3K (Ser164) and**	87

partly involves with AMPK-independent signaling [85]. Similarly, researches demonstrated that a dynamic reductive AdipoR1 expression in the heart and adiponectin concentration are contributive for the increased I/R injury responsiveness while sustained insulin therapy ameliorates myocardial reperfusion injury via increasing AMPK phosphorylation in diabetic Rattus norvegicus [86 , 87]. Though some researchers have put forward

**that adiponectin** preserves **the** hearts **from I/R injury by**	106

deterrent of

**inducible nitric oxide synthase (iNOS**) or **endothelial NO synthase (eNOS**), AMPK **and**	69

Akt [88,89], The deficiency of AMPK demonstrates limited impact on the antioxidative and antinitrative protection provided by adiponectin [90]. It is well established that both

**hypoxia-inducible factor-1α (HIF-1α**) and **HO-1**	112

serve as crucial transcriptional regulators in hypoxic cells and act as a primary role in sustaining 14 homeostasis in cell, further investigations have found that up-regulation of HIF-1α or HO-1 could increase adiponectin expression in diabetic mouse hearts and ultimately mitigate I/R injury [91,92]. In microRNA (miRNA) profile aspect, findings suggested that hypoadiponectinemia in diabetic Mus musculus remarkably increased miRNA-

**449b expression and downregulated Nrf-1 and Ucp3**	108

levels leading to excessive ROS generation and worse

myocardial I/R injury [93]. **Inhibition of miR** -200c-3p **and activation of** 66

the AdipoR2/STAT3 signaling induced by propofol post-conditioning alleviates diabetic myocardial I/R injury [94]. Last but not the least, the ablation of CDH13 abolished adiponectin's cardioprotective effects and increased infarct size similarly via disrupting the stimulation of a capital adiponectin signaling pathway concentrated on AMPK phosphorylation in myocardial I/R models [95]. Subsequently, the adiponectin performs its diverse functions primarily via the intricate binding mechanisms it exhibits with the AdipoR1/R2 or CDH13 receptors, and aforementioned changes of the pathophysiological mechanisms, including oxidative stress, miRNA, transcription factors, apoptosis, autophagy, and cellular signaling mentioned above, crucially regulate cardiac metabolism, affecting

myocardial I/R injury under **diabetes. 2** .2 Leptin **The** discovery **of** leptin, **the** 77

first adipokine synthesized and secreted by WAT encoded by

the ob gene, by American scholar **Jeffrey** M. **Friedman in 1994** 41

marked a significant turning point in the understanding of WAT [96]. This discovery transformed the perception of WAT

from a passive energy storage reservoir **to a dynamic endocrine organ** 26

with active regulatory functions in behavior and metabolism. It weights 16 kDa and consists of 167 amino acids, exhibits

a tertiary structure similar to that of cytokines with **long** 44

chains of helices [97]. The predominant subtype of leptin receptor (LEPR), has been confirmed as the extended form that located in the hypothalamic arcuate 15 nucleus, namely LEPRb [98]. Initially, LEPRb was deemed to be the functional receptor due to its 300 cytoplasmic residues that is longer in humans than in Mus musculus. This domain contains multiple motifs necessary for interacting with other proteins and initiating signaling pathway activation, such as the JAK-STAT3/5 and AMPK-acetyl- coenzyme A carboxylase axis [99,100]. Study has shown that leptin acts a crucial part as the primary sensory factor for energy storage inside the human as WAT communicates with the energy metabolism to the brain through the secretion of leptin, which in turn acts on hypothalamic neurons involved in regulating appetite to suppress hunger and increase energy expenditure [101,102]. A murine model deficient in leptin was first established in 1959, utilizing the ob/ob (leptin-deficient, caused by

a single autosomal recessive mutation on the obese gene located on chromosome 6) and db/db	2

(LEPR-deficient, caused by

a single autosomal mutation on the leptin receptor gene) Mus musculus. These models	2

have been extensively utilized in the past 20 years

for the advancement of myocardial I/R injury models in	1

T2DM [103]. Upon administering recombinant leptin to ob/ob Mus musculus, significant reductions were observed in their overall fat mass, resulting in a notable decline in food intake. Furthermore, the treatment effectively alleviated hyperglycemic and hyperinsulinemic conditions, exhibiting promising therapeutic effects in managing these metabolic abnormalities [104]. Moreover, a substantial difference is noted between Mus musculus of ob/ob and db/db genotypes and Mus musculus of wild-type genotypes in the context of post-ischemic myocardial injuries, which is attributable to multiple signal transduction pathways associated with autophagy, apoptosis, impaired insulin sensitivity, and other factors [105]. For instance, inhibition of mitsugumin 53 (MG53) E3 ligase activity mediated with MG53S255 phosphorylation [106], activation of PI3K/Akt pathway [107], down- 16 regulation of

**histone 3 lysine 9 acetylation through histone deacetylase**	114

[108] and suppression of

**HMGB1-RAGE (receptor for advanced glycation end products) axis**	29

[109] are all reported to be associated with attenuation of myocardial ischemia reperfusion injury. However, leptin itself yields inconsistent consequences on myocardial I/R injury. Prior research revealed

**that the expression of** leptin **in** both **the** serum and **heart** markedly **decreased**	111

during the initial stage following myocardial I/R injury,

followed by a gradual **increase during the reperfusion** phase [110]. **In** 92

murine models, pre-administration with leptin resulted in a decline in cardiac and serum inflammation, improvement in myocardial reperfusion damage, and potentially involves the increase of PI3K-Akt-Nuclear Factor kappa B expression as a protective mechanism [111 , 112]. During reperfusion, the manipulation of leptin caused a substantial reduction in infarction risk and a postponement

in the opening of the mitochondrial permeability transition pore (MPTP 6

), potentially

mediated by the PI3K/Akt and p44/42 mitogen-activated protein kinase 27

signaling transducer [113]. When compared to the aforementioned leptin-cardioprotective effects, respective clinical investigations have exhibited a significant correlation between diabetes and cardiovascular complications [114,115]. To a certain extent, leptin is a inflammatory activator related to endothelial dysfunction, neointimal hyperplasia, thrombogenesis, cardiac hypertrophic and pro-remodeling [116,117,118]. Fortunately, later research found that leptin resistance is recognized as a remarkable risk indicator for CVD rather to leptin deficiency [119]. Excessive leptin and impaired leptin signaling shift cardiac substrate energy metabolism (glucose replaced by free fatty acid), and then trigger massive accumulation of lipid which induces lipid toxicity, poor mitochondrial functions, and increased generation of ROS in I/R injury under diabetic condition [120,121]. Besides, insulin diminishes the storage of leptin and promotes its secretion 17 directly in a physiological context, while leptin impedes the secretion of insulin, decreases the production and accumulation of fat, enhances the sensitivity of insulin receptors, and ultimately establishes equilibrium between fat homeostasis and energy homeostasis. Disruption of this equilibrium may lead to metabolic disturbances, insulin and leptin resistance coexist in diabetes, obesity and CVD due to both of them share the same signal transduction pathways

such as protein tyrosine phosphatase 1B (PTP1B) and suppressor of cytokine signaling 3 41

(SOC3) [122,123]. What’s more, researchers suggested that targeting cannabinoid (CB) receptors/modulating the degree or activity of endocannabinoids in tissue is beneficial to decrease insulin/leptin resistance in diabetes and mitigate the myocardial damage during I/R phase [124 , 125]. Taken together, level of leptin could be identified

as a potential diagnostic **tool** and label **for** diabetic **myocardial I/R injury** 84

and managing appropriate leptin levels in individuals subjected to diabetes and myocardial I/R injury could be real fundamental for their metabolic well-being and holistic systemic health. 2.3 Resistin Resistin was initially identified for its role in promoting insulin resistance which controlled by the RETN gene in humans. Discovered in Mus musculus in 2001, this molecule is part of the resistin-like molecules family, characterized by

a unique cysteine repeat motif (C-X11-C-X8-C-X-C-X3-C-X10-C-X-C-X-C-X9-CC-X3-6-END 34

) and exhibiting diverse expression patterns and biological functions [126]. There is ongoing argumentation with respect to the effective impact of resistin in Mus musculus and Homo sapiens. Earlier research indicated that mouse resistin is embedded in chromosome 8 and weighing 11kDa, while Homo sapiens resistin is situated in chromosome 19 and weighing 12.5kDa, share 59% identity compared to the amino acid content, 64.4% sequence identity at the messenger RNA content, but only 46.7% 18 sequence identity at the DNA level [127]. Moreover, resistin is commonly generated by WAT in Mus musculus, whereas in Homo sapiens, macrophages are the main origination of resistin [128]. Despite the variances between humans and rodents, there is an increasing amount of demonstrations supporting that resistin acted as a mediator in the pathogenesis of inflammatory processes and the beginning

of several chronic diseases, such as metabolic malfunction, CVD, **and** 97

tumor [129]. On the one hand, increased contents of resistin have been described in instances of both diet-induced obese and genetically- induced obesity while treatment of anti-resistin agent has been found to against high glucose level and to mitigate insulin resistance in experimental animals with obesity in prior studies [130]. Nagaev and his co-authors contend that

there exists a dearth of correlation between insulin resistance, T2DM, **and** 109

resistin expression in both adipocytes and skeletal muscle. They have noted that while resistin expression is generally low in these tissues, it can still be detected in isolated adipocytes and total WAT from certain subjects [131]. Interestingly, recent study pointed out that high content of resistin is linked to escalated mortality in T2DM and expression level of greater than or equivalent to 11ng/mL indicates an elevated risk of poor outcomes [132]. It seems that

fatty acid transport protein 1 , CD36, AMPK, and **Acetyl-CoA carboxylase** 15

are related to insulin response and resistin function under hyperglycemia [133]. Despite that,

the existing data on the effects of resistin on **the** 31

myocardium have been inconclusive. Researchers demonstrated that resistin can distinctly reduce apoptotic rate and post-ischemic myocardial infarction area via PI3K/Akt/PKC or ERK1/2-matrix metalloproteinase 9 dependent pathways and thus against I/R injury [134,135]. By contrast, studies revealed that resistin yielded non- cardioprotective effects in Langendorff-perfused rodents hearts and lacking defence in human atrial muscle 19 subjected to reoxygenation damage [136] and resistin itself even worsens cardiac I/R injury through influencing the level of atrial natriuretic peptides during reperfusion and altering biochemical indicators of myocardial injury [137]. In addition, resistin exhibits the ability to react

to two distinct **receptors, toll-like receptor 4 (TLR4) and adenylyl cyclase-associated protein 1 (CAP1** 49

), thereby facilitating the initiation of inflammatory processes [138]. Currently, there is a lack of understanding regarding the potential cross-talk failure between diabetes and myocardial I/R injury. This discrepancy is connected with the significant disparities observed in the genetic and proteomic configuration of the resistin molecule between rodents and humans, and a scarcity of proof concerning the resistin receptor and its downstream signaling transducer pathways. Further assessment of the roles of resistin

in patients suffering from both diabetes **and** myocardial **I/R injury** 110

could improve our comprehension of the underlying pathways

involved in the physiological **and** pathological **development of** the **disease and** 90

potentially lead to improved treatment strategies for affected individuals. 2.4 Apelin In 1998, apelin was initially obtained from stomach extracts in bovine and characterized as a

ligand for the human **G protein-coupled receptor** (also referred to **apelin** receptor (**APJ**)) [139]. It distributes **in** 68

both human and mouse WAT, which is regulated by insulin and obesity [140]. Furthermore, apelin and APJ exhibit expression in different tissues, including but not limited to the cardiac tissue, lung, kidney, and tumor tissues, in addition to adipocytes [141].

Apelin peptides are produced by the enzymatic **cleavage of a 77-amino-acid precursor** molecule called **pre-pro-apelin** 25

at the C-terminal end. These peptides exhibit diverse lengths in the circulating system, undergoing sequential cleavage to generate shorter, less well-defined forms, with the predominant isoforms being

apelin-12, apelin -36, **apelin-17, and apelin** -13 [142]. Besides, **these** 107

subtypes 20 have the ability to bind APJ, however, conformational changes of receptors may impact protein function and effects [143]. And the preeminent physiologically active form of pyroglutamylated

apelin-13 is the dominant **apelin isoform** inside **the** circulatory **system and** Homo sapiens **plasma** 25

, with the capacity to potentially ameliorate vascular disease by inhibiting inflammation, suppressing apoptosis, reducing oxidative stress, and promoting autophagy [144,145]. Apelin signaling system is tie up with a range of physiological responses, which is known to contribute to multiple pathological conditions, notably cardiovascular disorders and diabetes. In a study by Kartal and his co-workers, apelin-13 was administrated in diabetic Rattus norvegicus before blood flow caseation of the coronary artery and the erythrocyte deformability was significantly increased [146]. Moreover, they found that apelin-13 inhibits cardiac cell death and excessive inflammation activity from I/R injury in diabetic rodents [147]. A latest study showed that apelin exhibits a protective outcome against I/R injury through suppressing apoptotic rate and ROS production via

the activation of PI3K and p38 **mitogen-activated protein kinase signaling** 86

in diabetic hearts [148]. Given its protective activities,

targeting the apelin for the treatment of CVD could be a 115

therapeutic tool and more studies should be conducted to further explore new potential mechanisms on diabetic heart combined with I/R injury. 2.5

Visfatin Visfatin, (also known as NAMPT – nicotinamide phosphoribosyltransferase or pre-B cell colony-enhancing factor (PBEF)) was 4

isolated from abdominal WAT by a Japanese research team in 2005, which is notably abundant in visceral fat tissue in Homo sapiens and Mus musculus, with its plasma expression extent rising accompanied by the progression of obesity and diabetes [149,150]. Prior researches demonstrated that increased content of visfatin in obese and diabetic subjects may help compensate for 21 prolonged high blood sugar by mimicking insulin and lowering glucose and lipid levels [151 , 152]. A cross-sectional multicentric study revealed that visfatin in diabetic patients who received drug treatment (such as angiotensin-converting-enzyme inhibitor, calcium channel blockers or statins) couldn't be used as a biomarker of subclinical atherosclerosis [153]. However, the function of visfatin in the physiological and pathological processes of diabetes is controversial. Prolonged visfatin treatment leading to a diabetic phenotype in Mus musculus [154] while another study reported that serum visfatin is linked to T2DM regardless of insulin resistance and obesity [155], indicating that the dual impact of visfatin on diabetes may be influenced by its concentration and requires further comprehensive clinical investigations. Furthermore, Sadoshima and colleagues conducted a series of studies examining the relation between serum levels of visfatin and I/R injury which indicated that prevention of visfatin downregulation can effectively inhibit apoptosis and stimulate autophagic flux in cardiac myocytes in response to prolonged ischemia and I/R by activating SIRT1 with upregulation of nicotinamide adenine dinucleotide (NAD+) and ATP contents [156,157,158]. Finally, Xin et al demonstrated that treatment of visfatin reduces the inflammation and apoptosis levels of myocardial cells after myocardial I/R through activation of PI3K/AKT/ heat shock protein 70 signaling axis [159] and latest study revealed that a circular RNA associated with ferroptosis mediates the visfatin-SIRT1-FoxO1-Fth1 signaling via regulating myocardial cell ferroptosis and preserving cardiac function during reperfusion injury [160]. Li et al suggested that activating the AMPK/NAMPT signaling improves the effectiveness

of sevoflurane post-conditioning in reducing myocardial I/R injury 118

, but this conclusion was later retracted due to inaccurate and incomplete data [161]. Off note,

1-(3,6-Dibromo-carbazol-9-yl)-3-phenylamino- propan-2-ol , known for 50

its ability to dimerize and aggregate visfatin, has been reported 22 to decrease the infarction area in diabetic hearts throughout cardiac ischemia and reperfusion damage recently, together

with molecular signaling modification **for p-AKT** , phosphorylated **eNOS and** 93

SIRT1 [162]. Straightforwardly, more in-depth investigations are vital

to examine the latent efficacy **of** visfatin **in** diabetic **myocardial I/R injury** 72

. 2.6 Adipsin, omentin, chemerin, and metrnl Adipokines discussed previously strongly correlate with diabetes and myocardial I/R injury. Moreover, other adipokines, including adipsin, omentin, chemerin and metrnl, have explicitly been linked to diabetes and myocardial I/R injury, respectively, providing valuable insights into the complex relationship between these two conditions. Additional research on these adipokines is necessary to support future in-depth clinical studies in this field. Adipsin,

known as complement factor D (CFD), was the **first** 12

adipokine discovered by Spiegelman's research team in 1987 and subsequently determined to be a serine protease homolog

produced and released by adipose **cells** , and **is** present **in the** 95

circulatory system [163]. Activated adipsin has minimal proteolytic activity on most substrates, but can cleave complement factor B as it bind to energized complement factor C3, its function is homologous to that of C1s in the classical pathway [164]. In comparison to Mus musculus, human adipsin messenger RNA is also observed in monocytes and macrophages [165]. Spiegelman's lab and others later found that diabetic individuals subjected to β cell failure are lacking adipsin expression [166] and adipsin/C3a preserves β cells via lowering the phosphatase DUSP26 in diabetic Mus musculus, potentially leading to beneficial effects that are linked to a decreased risk of developing T2DM in humans [167]. Meanwhile, adipsin mitigates mitochondrial damage and enhances β-oxidation of fatty acid in diabetic cardiomyopathy through its 23 interaction with Irak2 and impediment of Irak2 mitochondrial translocation [168]. Furthermore, exosomes originating from pericardial WAT mitigate post-myocardial infarction through adipsin-mediated regulation of iron homeostasis, while adipsin sourced from epicardial WAT contributes to

cardiomyocyte apoptosis after myocardial infarction via mediation of **PARP-1 activity** 13

[169,170]. Based on the findings of a cytokine array analysis, adipsin emerges as a novel biomarker with potential utility in forecasting re-hospitalization and mortality among individuals with coronary disease [171]. Despite the promising future perspectives of the aforementioned adipsin and adipsin compounds, there are currently no robust clinical treatments available that can effectively repair myocardial reperfusion injury in diabetic individuals. Omentin, metrnl, and chemerin are new adipokines discovered around 2005, which are mainly secreted by adipocytes to regulate the metabolism of adipocytes and exhibit either pro-inflammatory or anti-inflammatory properties in different clinical scenarios. Yang and colleagues discovered omentin (regulated by the ITLN1 gene) by analyzing

10,437 expressed sequence markers **from a human omental fat cDNA library** 33

which consists

of 313 amino acids and includes **a secretory signal sequence** as well as **a fibrinogen-related domain** [172]. **Omentin** exhibits high level **in** 33

omental adipose tissue, specifically in stromal vascular cells rather than adipocytes. Its molecular mechanisms contribute to protective effects on glucose homeostasis by mitigating inflammatory processes, improving insulin sensitivity, enhancing endothelial function, and facilitating vasodilation in obesity and diabetic subjects [173]. It confers cardioprotective benefits by mitigating

the progression of atherosclerosis **and heart failure** [174]. Furthermore, **the** systemic treatment **of** 89

human omentin in rodents resulted in a decline in myocardial infarction risk and apoptotic rate following I/R, concomitant with increased levels of AMPK and Akt in heart [175]. The precise role of omentin 24 remains uncertain at present; however, this molecule may serve as a noteworthy connection between diabetes and cardiovascular disease. Lastly,

metformin and statins could elevate omentin-1 levels 104

in patients [176], and thus, both of these medicines may be useful

in the treatment **of myocardial I/R injury under diabetic conditions. In** 1

2009, Surace et al identified and documented the presence of the metrnl (311 amino acid sequence encoded by 936 base pair sequence) gene on human chromosome 17 using bioinformatics analysis [177,178], it has become increasingly recognized as a high potential area of focus for investigation in a particular field of diabetes and CVD on recent years [179]. It

is highly expressed in the **skeletal muscle**, subcutaneous fatty **tissue**, epididymal WAT depots **and** 26

heart. Metrnl mitigates myocardial I/R injury- induced cardiomyocyte cell death by reducing over endoplasmic reticulum activity through the activation of AMPK-serine/threonine protein kinase pathway in cells [180]. Additionally, it improves diabetic cardiomyopathy by deactivating

Cyclic GMP-AMP Synthase-Stimulator of interferon genes signaling in **a** 28

manner dependent on Liver Kinase B1/AMPK/ UNC-51LikeAutophagyAckingKinase1-mediated autophagy [181]. Unfortunately,

the relation between serum content of metrnl **and the** danger **of heart disease** 19

in diabetic individuals remains inconclusive and contradictory, as evidenced by various controlled clinical trials or meta-analyses [182,183], suggesting that metrnl content could be affected by various elements. The limited understanding of its receptor or direct interacting proteins hinders further investigation of metrnl in myocardial damage,

both in the presence and absence of diabetes 14

. Regulated

by the gene **retinoic acid receptor responder protein 2 (RARRES2), chemerin is** mainly generated **by** 55

adipocytes to regulate the metabolism of adipocytes and exhibits proinflammatory and antiinflammatory properties through interaction with its main receptor, such as the

chemokine-like receptor 1 (CMKLR1), G protein - 25 **coupled receptor 1 (GPR1) and C-C chemokine receptor-like 2 (CCRL2** 30

) [184]. It is produced in an unactive precursor form known as prochemerin, the latter is released and subjected to proteolytic cleavage by diverse extracellular proteases, resulting in the generation of distinct isoforms exhibiting varying degrees of biological activity. Down- regulation of chemerin is beneficial to reduce reperfusion injury in response to intestinal, kidney, lung and brain damage, with the primary mechanisms being associated with NLRP3 inflammasome-mediated pyroptosis [185,186,187,188]. Proof based on human and animal studies revealed that dysregulation of chemerin may serve as a risk indicator for hyperglycemia, vascular inflammation, angiogenesis, atherosclerosis, chronic heart failure and blood pressure modulation [189].

Elevated levels of chemerin **have been linked to insulin resistance** 83

, disrupted blood glucose metabolism, and elevated blood glucose levels in Mus musculus [190]. Contrary to this, a recent study suggested that the addition of chemerin reversed cardiac dysfunction induced by lipid overload by increasing the messenger RNA levels of PPAR-γ

and PPAR-dependent genes (such as **CD36, Fabp4, and Fasn** 103

) and restoring the decrease in insulin-triggered Akt phosphorylation in Mus musculus treated with high-fat diet [191]. Additionally, a mendelian randomization study has identified potential associations between elevated genetically predicted levels of chemerin and a heightened risk of coronary disease [192]. The expanding number of research on chemerin's involvement in the pathological and physiological changes of CVD and diabetes has sparked interest in the potential use of chemerin and its associated signaling proteins as targets for the advancement of therapeutic medicines for the settlement of these conditions. 2.7 Potential interplay among various adipokines and receptors WAT is not merely a non-functional tissue but a complex and dynamic tissue that secretes adipokines in response to physiological and pathological stimuli. Due to its 26 intricate molecular signaling pathways, WAT is crucial in maintaining body homeostasis and exerts protective or damaging effects, thus motivating and continually expanding field of research. Prior studies have primarily focused on individual adipokines or their receptors in relation

to myocardial I/R injury in diabetes , rather than considering **in** 17

a broader perspective. Hence, we performed a network analysis of the aforementioned adipokines and receptors utilizing the STRING tools (http://cn.string- db.org) and Cytoscape software, aiming to outline potential interactions among adipokines in subjects who suffered myocardial I/R injury under hyperglycemia. Given the general constraint of

displaying no more than a maximum of 10 interactors, it is observed that metrnl has the fewest predicted functional partners compared to the other proteins (Fig. 3a-i). Moreover, resistin exhibits the highest anticipated number of edges, with adiponectin and leptin following closely behind (Table 1). Subsequently, we conducted a more in-depth examination of the correlation between adipokines and their respective receptors, resulting in the isolation of metrnl from the other proteins since the analysis is restricted to query proteins exclusively (Fig. 3j). Furthermore, a modular network was created applying the Cytohubba algorithm to reveal the core adipokine targets. The algorithm successfully identified significant network targets within the Protein-Protein Interaction (PPI) networks, with chemerin being the first target, followed by adiponectin, resistin, and visfatin.When considered collectively, adiponectin and resistin merit further investigation due to their significant potential and interconnected nature (Fig. 3k). However, this does not imply that metrnl is of lesser importance, as the PPI network is constructed using curated databases, experimentally determined data, protein homology, and other relevant factors. Fig. 3 The Protein-Protein Interaction (PPI) networks of potential targets of various adipokines. a. Networks of adiponectin (ADIPOQ); b. Networks of leptin (LEP); c. Networks of resistin (RETN); d. Networks of apelin (APLN); e. Networks of visfatin (NAMPT); f. Networks of adipsin (CFD); g. Networks of omentin (ITLN1); h. Networks of chemerin (RARRES2); i. Networks of metrnl (METRNL) (a-i: the orange oval represents adipokines, the green oval denotes non-self receptor proteins, and the purple oval signifies self receptor proteins); j. Networks of various adipokines and receptors; k. Ranking of adipokines and receptors (The darker node indicates a higher 663 ranking); (all PPI enrichment

p-value<0.05). 664 **Table** 1 665 The **results of the** 96

Protein-Protein Interaction (PPI) networks. Average local Expected Average Protein Gene Nodes Edges clustering number of node degree coefficient edges Adiponectin ADIPOQ 11 44 8 0.881 14 Leptin LEP 11 50 0.09 0.923 14 Resistin RETN 11 39 7.09 0.862 15 Apelin APLN 11 36 6.55 0.907 12 Visfatin NAMPT 11 52 9.45 0.958 11 Adipsin CFD 11 33 6 0.859 12 Omentin ITLN1 11 41 7.45 0.872 11 Chemerin RARRES2 11 37 6.73 0.796 11 Meteorin-like METRNL 6 11 3.67 0.933 5 protein 666 667 3. Limitation and considerations 668 According to empirical evidence, it is our contention that among the adipokines 669 identified thus far, adiponectin, leptin, resistin and apelin are the most favorable 670 contenders for clinical utilization, particularly in the context of myocardial protection 671 and diabetes. Yet, numerous potential avenues for further investigation persist, as the 672 existing data exhibit several inconsistencies. 673 Elevated levels of adiponectin, known for its against inflammatory and cardioprotective 674 effects, are linked to a declined risk of cardio damage in individuals with diabetes. Animal studies provide evidence supporting adiponectin as a cardioprotective protein in cardiovascular health [193], on the contrary, a two-year investigation found that heightened serum concentrations of adiponectin were linked to an increased likelihood of cardiovascular events leading to elevated mortality rates [194]. Besides, high- molecular-weight adiponectin is concerned with higher mortality in elder subjects compared with healthy middle-aged populations [195]. Furthermore, the expressed level of adiponectin receptors in human are influenced by gender, with males exhibiting significantly higher levels compared to females. Conversely, females demonstrate higher serum adiponectin levels than males [196]. Despite these conflicting associations, commonly referred to as the "adiponectin paradox" and previously elucidated factors such as renal dysfunction, adiponectin resistance, weight change, and hydrolysis of CDH13 [197,198], adiponectin still remain a major puzzle in the field. Therefore, it is utmost to further enhance our knowledge of adiponectin’s exact role and evaluate its potential in

order to achieve a balanced approach between minimizing diabetic myocardial I/R injury and mortality by specifically targeting cardiac adiponectin signaling pathway. Unlike other adipokines, adiponectin in blood does not seem to be affected by atorvastatin treatment in patients [199]. Lastly,

adiponectin levels were independently associated with restenosis , but both **HOMA-IR** 20

(Homeostatic

model assessment of insulin resistance) and adiponectin **were independently** associated **with** 78

de novo ischemic heart disease and the incidence of **new** percutaneous coronary interventions **in** patients **with normal glucose tolerance** 54

[200]. The ob/ob and db/db rodent genotypes serve as representations of diabetes as a monogenic disorder, contrasting with the polygenic and multifactorial nature of human T2DM [201]. For instance, hyperglycemia progresses gradually and deteriorates over time in humans, while blood glucose levels exhibit transient and limited severity in 30 ob/ob Mus musculus, with not all db/db Mus musculus experiencing the development of hyperglycemia [202,203]. Consequently, although these models are valuable for investigation purposes, the findings may have limited applicability, especially in diabetic myocardial I/R injury. Leptin's effects are currently incongruous and lacks comprehensive understanding, it is widely agreed upon that both elevated expression of leptin and leptin deficiency may have potential impacts for CVD. In-depth research endeavors are imperative in order to definitively ascertain whether these effects are being fine-tuned by distinct molecular signaling pathways or particular receptor isoforms. Certain hypoglycemic agents, including metformin and

sodium-glucose cotransporter 2 (SGLT2) inhibitors, have been documented **to** enhance **cardiac** 11

outcomes. Research indicates that metformin therapy, by reducing pericoronary fat levels, contributes to improved cardiovascular outcomes through the diminution of inflammatory markers, SGLT2, and leptin levels in individuals with pre-diabetes [204]. Furthermore, another study suggests that SGLT2 inhibitors may mitigate the inflammatory profile in patients with diabetes [205]. Take together, there appears to be a potential association between leptin and SGLT2. Due to the insufficient availability of reliable and comprehensive data on resistin, it cannot be considered as a reliable independent predictor of either diabetes or CVD. However, a clinical trial showed that levels of several adipokines significantly changed in individuals suffering

coronary artery bypass graft surgery with cardiopulmonary bypass 46

, with concentrations of adiponectin and adipsin diminished, but levels of leptin and resistin significantly augmented within 24 hours following the commencement of the operation [206]. Combination of several kinds of adipokines may act as a functional biomarker or risk predictor in I/R injury. Adiponectin-resistin (AR) index (fasting serum total adiponectin and resistin levels) and insulin resistance-AR (IRAR) index 31 (

integration of the AR index into an existing insulin resistance index) have been used **to** 18

screen individuals with elevated risk of potential progress of

T2DM and metabolic syndrome before [207], **recent study** found **that** both **of** 119

them applies on cardiovascular risk in diabetic patients as well [208]. The indices pertaining to AR and IRAR underwent a marked and significant upsurge in diabetic group compared with control group and further analysis demonstrated that these indices calculated level of cardiovascular risk through area under the curve [208]. Besides, adipose-derived stem cells (ADSCs) are regarded as potential instruments for the replacement, repair, and regeneration of necrotic or impaired cells [209]. He et al. utilized ADSCs

in a murine **model of I/R injury** , employing **both** resistin-treated **and** 76

vehicle-treated ADSCs. Their findings indicated that ADSCs treated with resistin significantly enhanced myocardial ejection fraction and reduced myocyte apoptosis [135]. ADSCs have been the subject of numerous

Phase I and II clinical trials , including **the** use **of** 100

a transendocardial delivery system for administering stromal vascular fraction to the akinetic myocardial scar region [210], as well as intra-articular injections of allogeneic ADSCs for the treatment of knee osteoarthritis [211]. Nonetheless, the intravenous administration of ADSCs has demonstrated limited retention and survival rates of myocardial stem cells [212]. Apelin

has been suggested as a novel **biomarker for** prognostication **in** myocardial ischemic 74

patients

with ST-segment elevation, with studies indicating that elevated plasma levels of apelin upon admission are in connection to a significant risk of mortality at the 6-month follow-up, thus augmenting the prognostic value provided by brain natriuretic peptide [213]. Certain researchers have posited that the endogenous release of the peptide may serve to mitigate the extent of an infarction [214] and the apelin/APJ system functions to mitigate imbalanced oxidative reaction between oxygen 32 and lipid in mitochondrial by facilitating the formation of nitric oxide during myocardial reperfusion damage as well [215]. Furthermore, the clinical utility of apelin as a therapeutic agent is restricted by its brief half-life and the requirement for parenteral delivery. Various studies have been undertaken to investigate potential small molecule apelin agonists, yet only a few have been progressed to further evaluation [216]. Given that Apelin has been shown to interplay with caveolin in cardiomyocytes [217], it is possible that apelin may interact with adiponectin via caveolin

in the context **of myocardial I/R injury in** diabetes, although further **study** is needed **to** 65

test this hypothesis. Conclusion Taken together, adipokines should be regarded as prospective therapeutic targets for CVD, necessitating further research on optimizing adipokine levels to mitigate the systemic impact of adipokines on

myocardial I/R injury in subjects **with diabetes. It is** 73

advisable to begin monitoring the dynamic changes of blood adipokines in diabetic patients, given that the current investigation of various adipokines lacks a comprehensive analysis of preclinical and clinical data. Meanwhile, much deeper research is necessary to investigate potential molecular mechanisms underlying the co- occurrence of diabetes and myocardial I/R injury, mainly focusing on the interaction between oxidative response, lipid imbalance, and programmed cell death pathways. Developing small-molecule adipokine compounds, including agonists and inhibitors or synthetic adipokine analogs, is recommended to facilitate future clinical studies in this area. 4. Reference [1] Martin SS, Aday AW, Almarzooq ZI, Anderson CAM, Arora P, Avery CL, et al. 33 American Heart Association Council on Epidemiology and Prevention Statistics Committee and Stroke Statistics Subcommittee. 2024 Heart Disease and Stroke Statistics: A Report of US and Global Data From the American Heart Association. Circulation. 2024 Feb 20;149(8):e347-e913. [2] Chang AJ, Liang Y, Hamilton SA, Ambrosy AP. Medical Decision-Making and Revascularization in Ischemic Cardiomyopathy. Med Clin North Am. 2024 May;108(3):553-566. [3] Di Gioia G, Soto Flores N, Franco D, Colaiori I, Sonck J, Gigante C, et al. Coronary Artery Bypass Grafting or Fractional Flow Reserve-Guided Percutaneous Coronary Intervention in Diabetic Patients With Multivessel Disease. Circ Cardiovasc Interv. 2020 Oct;13(10):e009157. [4] JENNINGS RB, SOMMERS HM, SMYTH GA, FLACK HA, LINN H. Myocardial necrosis induced by temporary occlusion of a coronary artery in the dog. Arch Pathol. 1960 Jul;70:68-78. [5] Heusch G. Myocardial ischemia/reperfusion: Translational pathophysiology of ischemic heart disease. Med. 2024 Jan 12;5(1):10-31. [6] Francisco J, Del Re DP. Inflammation in Myocardial Ischemia/Reperfusion Injury: Underlying Mechanisms and Therapeutic Potential. Antioxidants (Basel). 2023 Oct 31;12(11):1944. [7] GBD 2021 Diabetes Collaborators. Global, regional, and national

burden of diabetes from 1990 to 2021, with projections of prevalence to 2050: a systematic analysis for the Global Burden of Disease Study 2021. Lancet. 2023 Jul 15;402(10397):203-234. [8] Jiang Y, Cai Y, Han R, Xu Y, Xia Z, Xia W. Salvianolic acids and its potential for cardio-protection against myocardial ischemic reperfusion injury in diabetes. Front Endocrinol (Lausanne). 2024 Jan 12;14:1322474. [9] Babes EE, Bustea C, Behl T, Abdel-Daim MM, Nechifor AC, Stoicescu M, et al. Acute coronary syndromes in diabetic patients, outcome, revascularization, and antithrombotic therapy. Biomed Pharmacother. 2022 Apr;148:112772. [10] Pan J, Yin J, Gan L, Xue J. Two-sided roles of adipose tissue: Rethinking the obesity paradox in various human diseases from a new perspective. Obes Rev. 2023 Jan;24(1):e13521. [11] Oikonomou EK, Antoniades C. The role of adipose tissue in cardiovascular health and disease. Nat Rev Cardiol. 2019 Feb;16(2):83-99. 34 [12] Kaminska B, Kurowicka B, Kiezun M, Dobrzyn K, Kisielewska K, Gudelska M, et al. The Role of Adipokines in the Control of Pituitary Functions. Animals (Basel). 2024 Jan 22;14(2):353. [13] Xie L, Wang H, Hu J, Liu Z, Hu F. The role of novel adipokines and adipose- derived extracellular vesicles (ADEVs): Connections and interactions in liver diseases. Biochem Pharmacol. 2024 Apr;222:116104. [14] Polkinghorne MD, West HW, Antoniades C. Adipose Tissue in Cardiovascular Disease: From Basic Science to Clinical Translation. Annu Rev Physiol. 2024 Feb 12;86:175-198. [15] Akoumianakis I, Antoniades C. The interplay between adipose tissue and the cardiovascular system: is fat always bad? Cardiovasc Res. 2017 Jul 1;113(9):999-1008. [16] Semerena E, Nencioni A, Masternak K. Extracellular nicotinamide phosphoribosyltransferase: role in disease pathophysiology and as a biomarker. Front Immunol. 2023 Oct 17;14:1268756. [17] Yue H, Zhang Q, Chang S, Zhao X, Wang M, Li W. Adiponectin protects against myocardial ischemia-reperfusion injury: a systematic review and meta-analysis of preclinical animal studies. Lipids Health Dis. 2024 Feb 17;23(1):51. [18] Wang T, Yao S, Xia Z, Irwin MG. Adiponectin: mechanisms and new therapeutic approaches for restoring diabetic heart sensitivity to ischemic post-conditioning. Front Med. 2013 Sep;7(3):301-5. [19] Omoto ACM, do Carmo JM, Nelson B, Aitken N, Dai X, Moak S, et al. Central Nervous System Actions of Leptin Improve Cardiac Function After Ischemia- Reperfusion: Roles of Sympathetic Innervation and Sex Differences. J Am Heart Assoc. 2022 Nov;11(21):e027081. [20] Emerging Risk Factors Collaboration; Sarwar N, Gao P, Seshasai SR, Gobin R, Kaptoge S, et al. Diabetes mellitus, fasting blood glucose concentration, and risk of vascular disease: a collaborative meta-analysis of 102 prospective studies. Lancet. 2010 Jun 26;375(9733):2215-22. [21] Vuori MA, Reinikainen J, Söderberg S, Bergdahl E, Jousilahti P, Tunstall-Pedoe H, et al. Diabetes status-related differences in risk factors and mediators of heart failure in the general population: results from the MORGAM/BiomarCaRE consortium. Cardiovasc Diabetol. 2021 Sep 28;20(1):195. [22] Strain WD, Paldnnius PM. Diabetes, cardiovascular disease and th3e5 microcirculation. Cardiovasc Diabetol. 2018 Apr 18;17(1):57. [23] Henning RJ. Type-2 diabetes mellitus and cardiovascular disease. Future Cardiol. 2018 Nov;14(6):491-509. [24] Ansley DM, Wang B. Oxidative stress and myocardial injury in the diabetic heart. J Pathol. 2013 Jan;229(2):232-41. [25] Zhou J, Xia W, Chen J, Han K, Jiang Y, Zhang A, et al. Propofol and salvianolic acid A synergistically attenuated cardiac ischemia-reperfusion injury in diabetic mice via modulating the CD36/AMPK pathway. Burns Trauma. 2024 Apr 9;12:tkad055. [26] Han R, Huang H, Han H, Chen H, Zeng F, Xie X, et al. Propofol postconditioning ameliorates hypoxia/reoxygenation induced H9c2 cell apoptosis and autophagy via upregulating forkhead transcription factors under hyperglycemia. Mil Med Res. 2021 Nov 10;8(1):58. [27] Bai Y, Wu J, Yang Z, Wang X, Zhang D, Ma J. Mitochondrial quality control in cardiac ischemia/reperfusion injury: new insights into mechanisms and implications. Cell Biol Toxicol. 2023 Feb;39(1):33-51. [28] Munkhjargal U, Fukuda D, Maeda J, Hara T, Okamoto S, Bavuu O, et al. LCZ696, an Angiotensin Receptor-Neprilysin Inhibitor, Ameliorates Endothelial Dysfunction in Diabetic C57BL/6 Mice. J Atheroscler Thromb. 2024 Apr 13. [29] Bao XL, Dai Y, Lu L, Wang XQ, Ding FH, Shen WF, et al. Vasostatin-2 associates with coronary collateral vessel formation in diabetic patients and promotes angiogenesis via angiotensin-

converting enzyme 2. Eur Heart J. 2023 May 14;44(19):1732-1744. [30] Li X, Zou J, Lin A, Chi J, Hao H, Chen H, et al. Oxidative stress, endothelial dysfunction, and N-acetylcysteine in type-2 diabetes mellitus. Antioxid Redox Signal. 2024 Mar 18. [31]Gallo G, Savoia C. New Insights into Endothelial Dysfunction in Cardiometabolic Diseases: Potential Mechanisms and Clinical Implications. Int J Mol Sci. 2024 Mar 4;25(5):2973. [32] Russo I, Penna C, Musso T, Popara J, Alloatti G, Cavalot F, et al. Platelets, diabetes and myocardial ischemia/reperfusion injury. Cardiovasc Diabetol. 2017 May 31;16(1):71. [33] Ziegler M, Wang X, Peter K. Platelets in cardiac ischaemia/reperfusion injury: a promising therapeutic target. Cardiovasc Res. 2019 Jun 1;115(7):1178-1188. 36 [34] Manrique-Acevedo C, Hirsch IB, Eckel RH. Prevention of Cardiovascular Disease in Type 1 Diabetes. N Engl J Med. 2024 Apr 4;390(13):1207-1217. [35] Yang T, Zhang D. Research progress on the effects of novel hypoglycemic drugs in diabetes combined with myocardial ischemia/reperfusion injury. Ageing Res Rev. 2023 Apr;86:101884. [36] Asleh R, Sheikh-Ahmad M, Briasoulis A, Kushwaha SS. The influence of anti- hyperglycemic drug therapy on cardiovascular and heart failure outcomes in patients with type 2 diabetes mellitus. Heart Fail Rev. 2018 May;23(3):445-459. [37] ADVANCE Collaborative Group; Patel A, MacMahon S, Chalmers J, Neal B, Billot L, et al. Intensive blood glucose control and vascular outcomes in patients with type 2 diabetes. N Engl J Med. 2008 Jun 12;358(24):2560-72. [38] Patterson B, Fields AV, Shannon RP. New insights into myocardial glucose metabolism: surviving under stress. Curr Opin Clin Nutr Metab Care. 2009 Jul;12(4):424-30. [39] Tian H, Zhao X, Zhang Y, Xia Z. Abnormalities of glucose and lipid metabolism in myocardial ischemia-reperfusion injury. Biomed Pharmacother. 2023 Jul;163:114827. [40] Penna C, Andreadou I, Aragno M, Beauloye C, Bertrand L, Lazou A, et al. Effect of hyperglycaemia and diabetes on acute myocardial ischaemia-reperfusion injury and cardioprotection by ischaemic conditioning protocols. Br J Pharmacol. 2020 Dec;177(23):5312-5335. [41] Yellon DM, Beikoghli Kalkhoran S, Davidson SM. The RISK pathway leading to mitochondria and cardioprotection: how everything started. Basic Res Cardiol. 2023 May 26;118(1):22. [42] Wang T, Mao X, Li H, Qiao S, Xu A, Wang J, et al. N-Acetylcysteine and allopurinol up-regulated the Jak/STAT3 and PI3K/Akt pathways via adiponectin and attenuated myocardial postischemic injury in diabetes. Free Radic Biol Med. 2013 Oct;63:291-303. [43] Xue R, Lei S, Xia ZY, Wu Y, Meng Q, Zhan L, et al. Selective inhibition of PTEN preserves ischaemic post-conditioning cardioprotection in STZ-induced Type 1 diabetic rats: role of the PI3K/Akt and JAK2/STAT3 pathways. Clin Sci (Lond). 2016 Mar;130(5):377-92. [44] Gedik N, Kottenberg E, Thielmann M, Frey UH, Jakob H, Peters J, et al. Potenti3a7l humoral mediators of remote ischemic preconditioning in patients undergoing surgical coronary revascularization. Sci Rep. 2017 Oct 4;7(1):12660. [45] Heusch G, Musiolik J, Kottenberg E, Peters J, Jakob H, Thielmann M. STAT5 activation and cardioprotection by remote ischemic preconditioning in humans: short communication. Circ Res. 2012 Jan 6;110(1):111-5. [46] Huang Q, Tian H, Tian L, Zhao Xs, Li L, Zhang YX, et al. Inhibiting Rev-erbα- mediated ferroptosis alleviates susceptibility to myocardial ischemia-reperfusion injury in type 2 diabetes. Free Radic Biol Med. 2023 Nov 20;209(Pt 1):135-150. [47] Li W, Li W, Leng Y, Xiong Y, Xia Z. Ferroptosis Is Involved in Diabetes Myocardial Ischemia/Reperfusion Injury Through Endoplasmic Reticulum Stress. DNA Cell Biol. 2020 Feb;39(2):210-225. [48] Zhou D, Yang Y, Chen J, Zhou J, He J, Liu D, et al. N-acetylcysteine Protects Against Myocardial Ischemia-Reperfusion Injury Through Anti-ferroptosis in Type 1 Diabetic Mice. Cardiovasc Toxicol. 2024 Apr 22. [49] Ryabov VV, Maslov LN, Vyshlov EV, Mukhomedzyanov AV, Kilin M, Gusakova SV, et al. Ferroptosis, a Regulated Form of Cell Death, as a Target for the Development of Novel Drugs Preventing Ischemia/Reperfusion of Cardiac Injury, Cardiomyopathy and Stress-Induced Cardiac Injury. Int J Mol Sci. 2024 Jan 11;25(2):897. [50] Brennan S, Chen S, Makwana S, Martin CA, Sims MW, Alonazi ASA, et al. A novel form of glycolytic metabolism-dependent cardioprotection revealed by PKCα and β inhibition. J Physiol. 2019 Sep;597(17):4481-4501. [51] Zhang F, Cao X, Zhao C, Chen L, Chen X. Empagliflozin activates JAK2/STAT3 signaling and protects

cardiomyocytes from hypoxia/reoxygenation injury under high glucose conditions. J Thromb Thrombolysis. 2023 Jan;55(1):116-125. [52] Wang C, Zhu L, Yuan W, Sun L, Xia Z, Zhang Z, et al. Diabetes aggravates myocardial ischaemia reperfusion injury via activating Nox2-related programmed cell death in an AMPK-dependent manner. J Cell Mol Med. 2020 Jun;24(12):6670-6679. [53] Chen C, Lu C, He D, Na N, Wu Y, Luo Z, et al. Inhibition of HMGB1 alleviates myocardial ischemia/reperfusion injury in diabetic mice via suppressing autophagy. Microvasc Res. 2021 Nov;138:104204. [54] Zhang J, Cai X, Zhang Q, Li X, Li S, Ma J, et al. Hydrogen sulfide restores sevoflurane postconditioning mediated cardioprotection in diabetic rats: Role of SIRT1/Nrf2 signaling-modulated mitochondrial dysfunction and oxidative stress. J Ce3l8l Physiol. 2021 Jul;236(7):5052-5068. [55] Yang K, Velagapudi S, Akhmedov A, Kraler S, Lapikova-Bryhinska T, Schmiady MO, et al. Chronic SIRT1 supplementation in diabetic mice improves endothelial function by suppressing oxidative stress. Cardiovasc Res. 2023 Oct 16;119(12):2190- 2201. [56] Duan J, Guan Y, Mu F, Guo C, Zhang E, Yin Y, et al. Protective effect of butin against ischemia/reperfusion-induced myocardial injury in diabetic mice: involvement of the AMPK/GSK-3β/Nrf2 signaling pathway. Sci Rep. 2017 Jan 27;7:41491. [57] Gualillo O, Gonznlez-Juanatey JR, Lago F. The emerging role of adipokines as mediators of cardiovascular function: physiologic and clinical perspectives. Trends Cardiovasc Med. 2007 Nov;17(8):275-83. [58] Sato S. Adipo-oncology: adipocyte-derived factors govern engraftment, survival, and progression of metastatic cancers. Cell Commun Signal. 2024 Jan 18;22(1):52. [59] Sardu C, Pieretti G, D'Onofrio N, Ciccarelli F, Paolisso P, Passavanti MB, et al. Inflammatory Cytokines and SIRT1 Levels in Subcutaneous Abdominal Fat: Relationship With Cardiac Performance in Overweight Pre-diabetics Patients. Front Physiol. 2018 Aug 21;9:1030. [60] Krauz K, Kempiński M, Jańczak P, Momot K, Zarębiński M, Poprawa I, et al. The Role of Epicardial Adipose Tissue in Acute Coronary Syndromes, Post-Infarct Remodeling and Cardiac Regeneration. Int J Mol Sci. 2024 Mar 22;25(7):3583. [61] Liu Y, Dai L, Dong Y, Ma C, Cheng P, Jiang C, et al. Coronary inflammation based on pericoronary adipose tissue attenuation in type 2 diabetic mellitus: effect of diabetes management. Cardiovasc Diabetol. 2024 Mar 29;23(1):108. [62] Sardu C, Gatta G, Pieretti G, Viola L, Sacra C, Di Grezia G, et al. Pre-Menopausal Breast Fat Density Might Predict MACE During 10 Years of Follow-Up: The BRECARD Study. JACC Cardiovasc Imaging. 2021 Feb;14(2):426-438. [63] Scherer PE, Williams S, Fogliano M, Baldini G, Lodish HF. A novel serum protein similar to C1q, produced exclusively in adipocytes. J Biol Chem. 1995 Nov 10;270(45):26746-9. [64] Hu E, Liang P, Spiegelman BM. AdipoQ is a novel adipose-specific gene dysregulated in obesity. J Biol Chem. 1996 May 3;271(18):10697-703. [65] Maeda K, Okubo K, Shimomura I, Funahashi T, Matsuzawa Y, Matsubara K. cDNA cloning and expression of a novel adipose specific collagen-like factor, apM319 (AdiPose Most abundant Gene transcript 1). Biochem Biophys Res Commun. 1996 Apr 16;221(2):286-9. [66] Nakano Y, Tobe T, Choi-Miura NH, Mazda T, Tomita M. Isolation and characterization of GBP28, a novel gelatin-binding protein purified from human plasma. J Biochem. 1996 Oct;120(4):803-12. [67] Ouchi N, Shibata R, Walsh K. Cardioprotection by adiponectin. Trends Cardiovasc Med. 2006 Jul;16(5):141-6. [68] Goldstein BJ, Scalia RG, Ma XL. Protective vascular and myocardial effects of adiponectin. Nat Clin Pract Cardiovasc Med. 2009 Jan;6(1):27-35. [69] Ghadge AA, Khaire AA, Kuvalekar AA. Adiponectin: A potential therapeutic target for metabolic syndrome. Cytokine Growth Factor Rev. 2018 Feb;39:151-158. [70] Hug C, Wang J, Ahmad NS, Bogan JS, Tsao TS, Lodish HF. T-cadherin is a receptor for hexameric and high-molecular-weight forms of Acrp30/adiponectin. Proc Natl Acad Sci U S A. 2004 Jul 13;101(28):10308-13. [71] Siasos G, Tousoulis D, Kollia C, Oikonomou E, Siasou Z, Stefanadis C, et al. Adiponectin and cardiovascular disease: mechanisms and new therapeutic approaches. Curr Med Chem. 2012;19(8):1193-209. [72] Lim S, Quon MJ, Koh KK. Modulation of adiponectin as a potential therapeutic strategy. Atherosclerosis. 2014 Apr;233(2):721-728. [73] Barr LA, Shimizu Y, Lambert JP, Nicholson CK, Calvert JW. Hydrogen sulfide attenuates high fat diet-induced cardiac dysfunction via the suppression of

endoplasmic reticulum stress. Nitric Oxide. 2015 Apr 30;46:145-56. [74] Han X, Wu Y, Liu X, Ma L, Li T, Sun Q, et al. Adiponectin improves coronary no- reflow injury by protecting the endothelium in rats with type 2 diabetes mellitus. Biosci Rep. 2017 Jul 27;37(4):BSR20170282. [75] Tao L, Wang Y, Gao E, Zhang H, Yuan Y, Lau WB, et al. Adiponectin: an indispensable molecule in rosiglitazone cardioprotection following myocardial infarction. Circ Res. 2010 Feb 5;106(2):409-17. [76] Wang Y, Lau WB, Gao E, Tao L, Yuan Y, Li R, et al. Cardiomyocyte-derived adiponectin is biologically active in protecting against myocardial ischemia-reperfusion injury. Am J Physiol Endocrinol Metab. 2010 Mar;298(3):E663-70. [77] Rinaldi B, Di Filippo C, Capuano A, Donniacuo M, Sodano L, Ferraraccio F, et al. Adiponectin elevation by telmisartan ameliorates ischaemic myocardium in Zuck4e0r diabetic fatty rats with metabolic syndrome. Diabetes Obes Metab. 2012 Apr;14(4):320-8. [78] Wang T, Qiao S, Lei S, Liu Y, Ng KF, Xu A, et al. N-acetylcysteine and allopurinol synergistically enhance cardiac adiponectin content and reduce myocardial reperfusion injury in diabetic rats. PLoS One. 2011;6(8):e23967. [79] Cao C, Liu HM, Li W, Wu Y, Leng Y, Xue R, et al. Role of adiponectin in diabetes myocardial ischemia-reperfusion injury and ischemic postconditioning. Acta Cir Bras. 2020 Mar 23;35(1):e202000107. [80] Li H, Yao W, Liu Z, Xu A, Huang Y, Ma XL, et al. Hyperglycemia Abrogates Ischemic Postconditioning Cardioprotection by Impairing AdipoR1/Caveolin-3/STAT3 Signaling in Diabetic Rats. Diabetes. 2016 Apr;65(4):942-55. [81] Wang Y, Wang X, Jasmin JF, Lau WB, Li R, Yuan Y, et al. Essential role of caveolin-3 in adiponectin signalsome formation and adiponectin cardioprotection. Arterioscler Thromb Vasc Biol. 2012 Apr;32(4):934-42. [82] Sciarretta S, Frati G. The Importance of Restoring the Adiponectin Signaling Pathway to Reduce Myocardial Reperfusion Injury in Diabetes. Diabetes. 2016 Apr;65(4):826-8. [83] Meng Z, Zhang Z, Zhao J, Liu C, Yao P, Zhang L, et al. Nitrative Modification of Caveolin-3: A Novel Mechanism of Cardiac Insulin Resistance and a Potential Therapeutic Target Against Ischemic Heart Failure in Prediabetic Animals. Circulation. 2023 Apr 11;147(15):1162-1179. [84] Zhang Y, Zhao J, Li R, Lau WB, Yuan YX, Liang B, et al. AdipoRon, the first orally active adiponectin receptor activator, attenuates postischemic myocardial apoptosis through both AMPK-mediated and AMPK-independent signalings. Am J Physiol Endocrinol Metab. 2015 Aug 1;309(3):E275-82. [85] Wang Y, Liang B, Lau WB, Du Y, Guo R, Yan Z, et al. Restoring diabetes-induced autophagic flux arrest in ischemic/reperfused heart by ADIPOR (adiponectin receptor) activation involves both AMPK-dependent and AMPK-independent signaling. Autophagy. 2017;13(11):1855-1869. [86] Ma Y, Liu Y, Liu S, Qu Y, Wang R, Xia C, et al. Dynamic alteration of adiponectin/adiponectin receptor expression and its impact on myocardial ischemia/reperfusion in type 1 diabetic mice. Am J Physiol Endocrinol Metab. 2011 Sep;301(3):E447-55. 41 [87] Pei H, Qu Y, Lu X, Yu Q, Lian K, Liu P, et al. Cardiac-derived adiponectin induced by long-term insulin treatment ameliorates myocardial ischemia/reperfusion injury in type 1 diabetic mice via AMPK signaling. Basic Res Cardiol. 2013 Jan;108(1):322. [88] Tao L, Gao E, Jiao X, Yuan Y, Li S, Christopher TA, et al. Adiponectin cardioprotection after myocardial ischemia/reperfusion involves the reduction of oxidative/nitrative stress. Circulation. 2007 Mar 20;115(11):1408-16. [89] Gonon AT, Widegren U, Bulhak A, Salehzadeh F, Persson J, Sjöquist PO, et al. Adiponectin protects against myocardial ischaemia-reperfusion injury via AMP- activated protein kinase, Akt, and nitric oxide. Cardiovasc Res. 2008 Apr 1;78(1):116- 22. [90] Wang Y, Gao E, Tao L, Lau WB, Yuan Y, Goldstein BJ, et al. AMP-activated protein kinase deficiency enhances myocardial ischemia/reperfusion injury but has minimal effect on the antioxidant/antinitrative protection of adiponectin. Circulation. 2009 Feb 17;119(6):835-44. [91] Natarajan R, Salloum FN, Fisher BJ, Kukreja RC, Fowler AA 3rd. Hypoxia inducible factor-1 upregulates adiponectin in diabetic mouse hearts and attenuates post- ischemic injury. J Cardiovasc Pharmacol. 2008 Feb;51(2):178-87. [92] L'Abbate A, Neglia D, Vecoli C, Novelli M, Ottaviano V, Baldi S, et al. Beneficial effect of heme oxygenase-1 expression on myocardial ischemia-reperfusion involves an increase in adiponectin in mildly diabetic rats. Am J Physiol Heart Circ Physiol. 2007

Dec;293(6):H3532-41. [93] Meng Z, Liang B, Wu Y, Liu C, Wang H, Du Y, et al. Hypoadiponectinemia- induced upregulation of microRNA449b downregulating Nrf-1 aggravates cardiac ischemia-reperfusion injury in diabetic mice. J Mol Cell Cardiol. 2023 Sep;182:1-14. [94] Huang L, Ding L, Yu S, Huang X, Ren Q. Propofol postconditioning alleviates diabetic myocardial ischemia-reperfusion injury via the miR-200c-3p/AdipoR2/STAT3 signaling pathway. Mol Med Rep. 2022 Apr;25(4):137. [95] Denzel MS, Scimia MC, Zumstein PM, Walsh K, Ruiz-Lozano P, et al. T-cadherin is critical for adiponectin-mediated cardioprotection in mice. J Clin Invest. 2010 Dec;120(12):4342-52. [96] Zhang Y, Proenca R, Maffei M, Barone M, Leopold L, Friedman JM. Positional cloning of the mouse obese gene and its human homologue. Nature. 1994 Dec 1;372(6505):425-32. 42 [97] Friedman JM, Mantzoros CS. 20 years of leptin: from the discovery of the leptin gene to leptin in our therapeutic armamentarium. Metabolism. 2015 Jan;64(1):1-4. [98] Misch M, Puthanveetil P. The Head-to-Toe Hormone: Leptin as an Extensive Modulator of Physiologic Systems. Int J Mol Sci. 2022 May 13;23(10):5439. [99] Bjørbaek C, Uotani S, da Silva B, Flier JS. Divergent signaling capacities of the long and short isoforms of the leptin receptor. J Biol Chem. 1997 Dec 19;272(51):32686-95. [100] Frühbeck G. Intracellular signalling pathways activated by leptin. Biochem J. 2006 Jan 1;393(Pt 1):7-20. [101] Elmquist JK, Elias CF, Saper CB. From lesions to leptin: hypothalamic control of food intake and body weight. Neuron. 1999 Feb;22(2):221-32. [102] Fischer AW, Cannon B, Nedergaard J. Leptin: Is It Thermogenic? Endocr Rev. 2020 Apr 1;41(2):232–60. [103] Stachura A, Khanna I, Krysiak P, Paskal W, Włodarski P. Wound Healing Impairment in Type 2 Diabetes Model of Leptin-Deficient Mice-A Mechanistic Systematic Review. Int J Mol Sci. 2022 Aug 3;23(15):8621. [104] Hamann A, Matthaei S. Regulation of energy balance by leptin. Exp Clin Endocrinol Diabetes. 1996;104(4):293-300. [105] Greer JJ, Ware DP, Lefer DJ. Myocardial infarction and heart failure in the db/db diabetic mouse. Am J Physiol Heart Circ Physiol. 2006 Jan;290(1):H146-53. [106] Lv F, Wang Y, Shan D, Guo S, Chen G, Jin L, et al. Blocking MG53S255 Phosphorylation Protects Diabetic Heart From Ischemic Injury. Circ Res. 2022 Dec 2;131(12):962-976. [107] Sun D, Li S, Wu H, Zhang M, Zhang X, Wei L, et al. Oncostatin M (OSM) protects against cardiac ischaemia/reperfusion injury in diabetic mice by regulating apoptosis, mitochondrial biogenesis and insulin sensitivity. J Cell Mol Med. 2015 Jun;19(6):1296-307. [108] Huang G, Cheng Z, Hildebrand A, Wang C, Cimini M, Roy R, et al. Diabetes impairs cardioprotective function of endothelial progenitor cell-derived extracellular vesicles via H3K9Ac inhibition. Theranostics. 2022 May 21;12(9):4415-4430. [109] He D, Liu D, Luo X, Chen C, Lu C, Na N, et al. HMGB1-RAGE axis contributes to myocardial ischemia/reperfusion injury via regulation of cardiomyocyte autophagy and apoptosis in diabetic mice. Biol Chem. 2023 Sep 28;405(3):167-176. 43 [110] Xue H, Yan G, Lin J, Hao X. Preliminary investigation of the changes and mechanism of Leptin after myocardial ischemia/reperfusion injury. Zhongguo Wei Zhong Bing Ji Jiu Yi Xue. 2010 Nov;22(11):680-3. [111] Xu T, Liu S, Wang X. Amelioration of myocardial ischemia/reperfusion injury by leptin pretreatment and ischemic preconditioning in mouse. Zhongguo Wei Zhong Bing Ji Jiu Yi Xue. 2010 Feb;22(2):105-8. [112] Xu S, Tao D. Leptin Alleviates Inflammatory Response in Myocardial Ischemia Reperfusion Injury. Dis Markers. 2022 Mar 9;2022:8707061. [113] Smith CC, Mocanu MM, Davidson SM, Wynne AM, Simpkin JC, Yellon DM. Leptin, the obesity-associated hormone, exhibits direct cardioprotective effects. Br J Pharmacol. 2006 Sep;149(1):5-13. [114] Sweeney G. Cardiovascular effects of leptin. Nat Rev Cardiol. 2010 Jan;7(1):22- 9. [115] Mitsis A, Kadoglou NPE, Lambadiari V, Alexiou S, Theodoropoulos KC, Avraamides P, et al. Prognostic role of inflammatory cytokines and novel adipokines in acute myocardial infarction: An updated and comprehensive review. Cytokine. 2022 May;153:155848. [116] Knudson JD, Payne GA, Borbouse L, Tune JD. Leptin and mechanisms of endothelial dysfunction and cardiovascular disease. Curr Hypertens Rep. 2008 Dec;10(6):434-9. [117] Karmazyn M, Gan XT. Molecular and Cellular Mechanisms Underlying the Cardiac Hypertrophic and Pro-Remodelling Effects of Leptin. Int J Mol Sci. 2024 Jan 17;25(2):1137. [118] Konstantinides S, Schäfer K,

Koschnick S, Loskutoff DJ. Leptin-dependent platelet aggregation and arterial thrombosis suggests a mechanism for atherothrombotic disease in obesity. J Clin Invest. 2001 Nov;108(10):1533-40. [119] Smith CC, Yellon DM. Adipocytokines, cardiovascular pathophysiology and myocardial protection. Pharmacol Ther. 2011 Feb;129(2):206-19. [120] Senesi P, Luzi L, Terruzzi I. Adipokines, Myokines, and Cardiokines: The Role of Nutritional Interventions. Int J Mol Sci. 2020 Nov 8;21(21):8372. [121] Vilariño-García T, Polonio-Gonznlez ML, Pérez-Pérez A, Ribalta J, Arrieta F, Aguilar M, et al. Role of Leptin in Obesity, Cardiovascular Disease, and Type 2 Diabetes. Int J Mol Sci. 2024 Feb 16;25(4):2338. 44 [122] Pedroso JAB, Silva IBD, Zampieri TT, Totola LT, Moreira TS, Taniguti APT, et al. SOCS3 Ablation in Leptin Receptor-Expressing Cells Causes Autonomic and Cardiac Dysfunctions in Middle-Aged Mice despite Improving Energy and Glucose Metabolism. Int J Mol Sci. 2022 Jun 10;23(12):6484. [123] Bhavana, Kohal R, Kumari P, Das Gupta G, Kumar Verma S. Druggable targets of protein tyrosine phosphatase Family, viz. PTP1B, SHP2, Cdc25, and LMW-PTP: Current scenario on medicinal Attributes, and SAR insights. Bioorg Chem. 2024 Mar;144:107121. [124] Pacher P, Haskó G. Endocannabinoids and cannabinoid receptors in ischaemia- reperfusion injury and preconditioning. Br J Pharmacol. 2008 Jan;153(2):252-62. [125] Pacher P, Steffens S. The emerging role of the endocannabinoid system in cardiovascular disease. Semin Immunopathol. 2009 Jun;31(1):63-77. [126] Steppan CM, Brown EJ, Wright CM, Bhat S, Banerjee RR, Dai CY, et al A family of tissue-specific resistin-like molecules. Proc Natl Acad Sci U S A. 2001 Jan 16;98(2):502-6. [127] Ghosh S, Singh AK, Aruna B, Mukhopadhyay S, Ehtesham NZ. The genomic organization of mouse resistin reveals major differences from the human resistin: functional implications. Gene. 2003 Feb 13;305(1):27-34. [128] Patel L, Buckels AC, Kinghorn IJ, Murdock PR, Holbrook JD, Plumpton C, et al. Resistin is expressed in human macrophages and directly regulated by PPAR gamma activators. Biochem Biophys Res Commun. 2003 Jan 10;300(2):472-6. [129] Jamaluddin MS, Weakley SM, Yao Q, Chen C. Resistin: functional roles and therapeutic considerations for cardiovascular disease. Br J Pharmacol. 2012 Feb;165(3):622-32. [130] Steppan CM, Bailey ST, Bhat S, Brown EJ, Banerjee RR, Wright CM, Patel HR, Ahima RS, Lazar MA. The hormone resistin links obesity to diabetes. Nature. 2001 Jan 18;409(6818):307-12. [131] Nagaev I, Smith U. Insulin resistance and type 2 diabetes are not related to resistin expression in human fat cells or skeletal muscle. Biochem Biophys Res Commun. 2001 Jul 13;285(2):561-4. [132] Kapłon-Cieślicka A, Tymińska A, Rosiak M, Ozierański K, Peller M, Eyileten C, et al. Resistin is a prognostic factor for death in type 2 diabetes. Diabetes Metab Res Rev. 2019 Feb;35(2):e3098. 45 [133] Saeedi Borujeni MJ, Esfandiary E, Taheripak G, Codoñer-Franch P, Alonso- Iglesias E, Mirzaei H. Molecular aspects of diabetes mellitus: Resistin, microRNA, and exosome. J Cell Biochem. 2018 Feb;119(2):1257-1272. [134] Gao J, Chang C, Chen Z, Wang H, Xu X, C Hamdy R, et al. Resistin, an adipocytokine, offers protection against acute myocardial infarction. J Mol Cell Cardiol. 2007 Nov;43(5):601-9. [135] He Y, Guo Y, Xia Y, Guo Y, Wang R, Zhang F, et al. Resistin promotes cardiac homing of mesenchymal stem cells and functional recovery after myocardial ischemia- reperfusion via the ERK1/2-MMP-9 pathway. Am J Physiol Heart Circ Physiol. 2019 Jan 1;316(1):H233-H244. [136] Smith CC, Lim SY, Wynne AM, Sivaraman V, Davidson SM, Mocanu MM, et al. Failure of the adipocytokine, resistin, to protect the heart from ischemia-reperfusion injury. J Cardiovasc Pharmacol Ther. 2011 Mar;16(1):63-71. [137] Rothwell SE, Richards AM, Pemberton CJ. Resistin worsens cardiac ischaemia- reperfusion injury. Biochem Biophys Res Commun. 2006 Oct 13;349(1):400-7. [138] Li C, Sun XN, Zhao S, Scherer PE. Crosstalk Between Adipose Tissue and the Heart: An Update. J Transl Int Med. 2022 Sep 24;10(3):219-226. [139] Tatemoto K, Hosoya M, Habata Y, Fujii R, Kakegawa T, Zou MX, et al. Isolation and characterization of a novel endogenous peptide ligand for the human APJ receptor. Biochem Biophys Res Commun. 1998 Oct 20;251(2):471-6. [140] Boucher J, Masri B, Daviaud D, Gesta S, Guigné C, Mazzucotelli A, et al. Apelin, a newly identified adipokine up-regulated by insulin and obesity. Endocrinology. 2005 Apr;146(4):1764-71. [141] Huang Z, Luo X,

Liu M, Chen L. Function and regulation of apelin/APJ system in digestive physiology and pathology. J Cell Physiol. 2019 Jun;234(6):7796-7810. [142] Hosoya M, Kawamata Y, Fukusumi S, Fujii R, Habata Y, Hinuma S, et al. Molecular and functional characteristics of APJ. Tissue distribution of mRNA and interaction with the endogenous ligand apelin. J Biol Chem. 2000 Jul 14;275(28):21061-7. [143] Wen R, Huang R, Xu K, Cheng Y, Yi X. Beneficial effects of Apelin-13 on metabolic diseases and exercise. Front Endocrinol (Lausanne). 2023 Nov 28;14:1285788. [144] Yamaleyeva LM, Shaltout HA, Varagic J. Apelin-13 in blood pressure regulatio4n6 and cardiovascular disease. Curr Opin Nephrol Hypertens. 2016 Sep;25(5):396-403. [145] Zeng G, Tang S, Jiang W, Yu J, Nie GY, Tang CK. Apelin-13: A Protective Role in Vascular Diseases. Curr Probl Cardiol. 2024 Jan;49(1 Pt B):102088. [146] Kartal H, Comu FM, Kucuk A, Polat Y, Dursun AD, Arslan M. Effect of apelin- 13 on erythrocyte deformability during ischaemia-reperfusion injury of heart in diabetic rats. Bratisl Lek Listy. 2017;118(3):133-136. [147] Gunes I, Kartal H, Dursun AD, Sungu N, Polat YS, Erkent FD, et al. Effects of apelin-13 on myocardial ischemia reperfusion injury in streptozotocine induced diabetic rats. Bratisl Lek Listy. 2018;119(6):348-354. [148] An S, Wang X, Shi H, Zhang X, Meng H, Li W, et al. Apelin protects against ischemia-reperfusion injury in diabetic myocardium via inhibiting apoptosis and oxidative stress through PI3K and p38-MAPK signaling pathways. Aging (Albany NY). 2020 Dec 20;12(24):25120-25137. [149] Chang Y, Chang D, Lin K, Shin S, Lee Y. Visfatin in overweight/obesity, type 2 diabetes mellitus, insulin resistance, metabolic syndrome and cardiovascular diseases: a meta-analysis and systemic review. Diabetes Metab Res Rev. 2011 Sep;27(6):515-27. [150] Fukuhara A, Matsuda M, Nishizawa M, Segawa K, Tanaka M, Kishimoto K, wt al. Visfatin: a protein secreted by visceral fat that mimics the effects of insulin. Science. 2005 Jan 21;307(5708):426-30. [151] Unlütürk U, Harmanci A, Yildiz BO, Bayraktar M. Dynamics of Nampt/visfatin and high molecular weight adiponectin in response to oral glucose load in obese and lean women. Clin Endocrinol (Oxf). 2010 Apr;72(4):469-74. [152] Katsareli EA, Dedoussis GV. Biomarkers in the field of obesity and its related comorbidities. Expert Opin Ther Targets. 2014 Apr;18(4):385-401. [153] Kärberg K, Forbes A, Lember M. Visfatin and Subclinical Atherosclerosis in Type 2 Diabetes: Impact of Cardiovascular Drugs. Medicina (Kaunas). 2023 Jul 18;59(7):1324. [154] Kieswich J, Sayers SR, Silvestre MF, Harwood SM, Yaqoob MM, Caton PW. Monomeric eNAMPT in the development of experimental diabetes in mice: a potential target for type 2 diabetes treatment. Diabetologia. 2016 Nov;59(11):2477-2486. [155] Esteghamati A, Alamdari A, Zandieh A, Elahi S, Khalilzadeh O, Nakhjavani M, et al. Serum visfatin is associated with type 2 diabetes mellitus independent of insulin resistance and obesity. Diabetes Res Clin Pract. 2011 Feb;91(2):154-8. 47 [156] Hsu CP, Oka S, Shao D, Hariharan N, Sadoshima J. Nicotinamide phosphoribosyltransferase regulates cell survival through NAD+ synthesis in cardiac myocytes. Circ Res. 2009 Aug 28;105(5):481-91. [157] Yamamoto T, Byun J, Zhai P, Ikeda Y, Oka S, Sadoshima J. Nicotinamide mononucleotide, an intermediate of NAD+ synthesis, protects the heart from ischemia and reperfusion. PLoS One. 2014 Jun 6;9(6):e98972. [158] Hsu CP, Yamamoto T, Oka S, Sadoshima J. The function of nicotinamide phosphoribosyltransferase in the heart. DNA Repair (Amst). 2014 Nov;23:64-8. [159] Xin B, Li P, Liu XL, Zhang XF. Visfatin relieves myocardial ischemia-reperfusion injury through activation of PI3K/Akt/HSP70 signaling axis. Eur Rev Med Pharmacol Sci. 2020 Oct;24(20):10779-10789. [160] Ju J, Li X, Zhao X, Li F, Wang S, Wang K, et al. Circular RNA FEACR inhibits ferroptosis and alleviates myocardial ischemia/reperfusion injury by interacting with NAMPT. J Biomed Sci. 2023 Jun 27;30(1):45. [161] Li T, Yu S, Zhou C, Wang K, Wan YC. MicroRNA-206 inhibition and activation of the AMPK/Nampt signalling pathway enhance sevoflurane post-conditioning- induced amelioration of myocardial ischaemia/reperfusion injury. J Drug Target. 2020 Jan;28(1):80-91. [162] Tur J, Badole SL, Manickam R, Chapalamadugu KC, Xuan W, Guida W, et al. Cardioprotective Effects of 1-(3,6-Dibromo-carbazol-9-yl)-3-Phenylamino-Propan-2- Ol in Diabetic Hearts via Nicotinamide Phosphoribosyltransferase Activation. J Pharmacol Exp Ther. 2022 Aug;382(2):233-245. [163] Flier JS, Cook KS, Usher

P, Spiegelman BM. Severely impaired adipsin expression in genetic and acquired obesity. Science. 1987 Jul 24;237(4813):405-8. [164] Rosen BS, Cook KS, Yaglom J, Groves DL, Volanakis JE, Damm D, et al. Adipsin and complement factor D activity: an immune-related defect in obesity. Science. 1989 Jun 23;244(4911):1483-7. [165] White RT, Damm D, Hancock N, Rosen BS, Lowell BB, Usher P, et al. Human adipsin is identical to complement factor D and is expressed at high levels in adipose tissue. J Biol Chem. 1992 May 5;267(13):9210-3. [166] Lo JC, Ljubicic S, Leibiger B, Kern M, Leibiger IB, Moede T, et al. Adipsin is an adipokine that improves β cell function in diabetes. Cell. 2014 Jul 3;158(1):41-53. [167] Gómez-Banoy N, Guseh JS, Li G, Rubio-Navarro A, Chen T, Poirier B, et a4l8. Adipsin preserves beta cells in diabetic mice and associates with protection from type 2 diabetes in humans. Nat Med. 2019 Nov;25(11):1739-1747. [168] Jiang M, Man W, Zhang X, Zhang X, Duan Y, Lin J, et al. Adipsin inhibits Irak2 mitochondrial translocation and improves fatty acid β-oxidation to alleviate diabetic cardiomyopathy. Mil Med Res. 2023 Dec 11;10(1):63. [169] Man W, Song X, Xiong Z, Gu J, Lin J, Gu X, et al. Exosomes derived from pericardial adipose tissues attenuate cardiac remodeling following myocardial infarction by Adipsin-regulated iron homeostasis. Front Cardiovasc Med. 2022 Sep 12;9:1003282. [170] Hao S, Zhang J, Pei Y, Guo L, Liang Z. Complement factor D derived from epicardial adipose tissue participates in cardiomyocyte apoptosis after myocardial infarction by mediating PARP-1 activity. Cell Signal. 2023 Jan;101:110518. [171] Ohtsuki T, Satoh K, Shimizu T, Ikeda S, Kikuchi N, Satoh T, et al. Identification of Adipsin as a Novel Prognostic Biomarker in Patients With Coronary Artery Disease. J Am Heart Assoc. 2019 Dec 3;8(23):e013716. [172] Yang RZ, Lee MJ, Hu H, Pray J, Wu HB, Hansen BC, et al Identification of omentin as a novel depot-specific adipokine in human adipose tissue: possible role in modulating insulin action. Am J Physiol Endocrinol Metab. 2006 Jun;290(6):E1253-61. [173] Sena CM. Omentin: A Key Player in Glucose Homeostasis, Atheroprotection, and Anti-Inflammatory Potential for Cardiovascular Health in Obesity and Diabetes. Biomedicines. 2024 Jan 26;12(2):284. [174] Vasamsetti SB, Natarajan N, Sadaf S, Florentin J, Dutta P. Regulation of cardiovascular health and disease by visceral adipose tissue-derived metabolic hormones. J Physiol. 2023 Jun;601(11):2099-2120. [175] Kataoka Y, Shibata R, Ohashi K, Kambara T, Enomoto T, Uemura Y, et al. Omentin prevents myocardial ischemic injury through AMP-activated protein kinase- and Akt-dependent mechanisms. J Am Coll Cardiol. 2014 Jun 24;63(24):2722-33. [176] Lin S, Li X, Zhang J, Zhang Y. Omentin-1: Protective impact on ischemic stroke via ameliorating atherosclerosis. Clin Chim Acta. 2021 Jun;517:31-40. [177] Surace C, Piazzolla S, Sirleto P, Digilio MC, Roberti MC, Lombardo A, et al. Mild ring 17 syndrome shares common phenotypic features irrespective of the chromosomal breakpoints location. Clin Genet. 2009 Sep;76(3):256-62. [178] Li Z, Gao Z, Sun T, Zhang S, Yang S, Zheng M, et al. Meteorin-like/Metrnl,4a9 novel secreted protein implicated in inflammation, immunology, and metabolism: A comprehensive review of preclinical and clinical studies. Front Immunol. 2023 Feb 24;14:1098570. [179] Miao Z, Hu W, Li Z, Miao C. Involvement of the secreted protein Metrnl in human diseases. Acta Pharmacol Sin. 2020 Dec;41(12):1525-1530. [180] Xu L, Cai Y, Wang Y, Xu C. Meteorin-Like (METRNL) Attenuates Myocardial Ischemia/Reperfusion Injury-Induced Cardiomyocytes Apoptosis by Alleviating Endoplasmic Reticulum Stress via Activation of AMPK-PAK2 Signaling in H9C2 Cells. Med Sci Monit. 2020 Jun 28;26:e924564. [181] Lu Q, Ding Y, Liu Y, Wang Z, Wu Y, Niu K, et al. Metrnl ameliorates diabetic cardiomyopathy via inactivation of cGAS/STING signaling dependent on LKB1/AMPK/ULK1-mediated autophagy. J Adv Res. 2023 Sep;51:161-179. [182] Liu Z, Ji H, Yao M, Wang L, Wang Y, Zhou P, et al. Serum Metrnl is associated with the presence and severity of coronary artery disease. J Cell Mol Med. 2019 Jan;23(1):271-280. [183] Ferns GA, Fekri K, Shahini Shams Abadi M, Banitalebi Dehkordi M, Arjmand MH. A meta-analysis of the relationship between serums metrnl-like protein/subfatin and risk of type 2 diabetes mellitus and coronary artery disease. Arch Physiol Biochem. 2023 Oct;129(5):1084-1090. [184] Lavis P, Bondue B, Cardozo AK. The Dual Role of Chemerin in Lung Diseases. Cells. 2024 Jan 16;13(2):171. [185] Liu L, Zhang J, Lu K, Zhang Y, Xu X, Deng

J,et al. ChemR23 signaling ameliorates brain injury via inhibiting NLRP3 inflammasome-mediated neuronal pyroptosis in ischemic stroke. J Transl Med. 2024 Jan 4;22(1):23. [186] Peng X, Wang W, Wang W, Qi J. Alpha-NETA, as a CMKLR1 Small Molecule Antagonist, Protects against Renal Ischemia Reperfusion Injury in Mice. Protein Pept Lett. 2022;29(11):962-970. [187] Zhu Q, He G, Li H. Effect of Intestinal Ischemia-reperfusion Injury on the Expression of Chemerin in Mice] Zhongguo Yi Xue Ke Xue Yuan Xue Bao. 2015 Aug;37(4):440-5. [188] Zou R, Wang M, Chen Y, Fan X, Yang B, Du J, et al. Hydrogen-Rich Saline Attenuates Acute Lung Injury Induced by Limb Ischemia/Reperfusion via Down-Regulating Chemerin and NLRP3 in Rats. Shock. 2019 Jul;52(1):134-141. 50 [189] Macvanin MT, Rizzo M, Radovanovic J, Sonmez A, Paneni F, Isenovic ER. Role of Chemerin in Cardiovascular Diseases. Biomedicines. 2022 Nov 18;10(11):2970. [190] Ernst MC, Issa M, Goralski KB, Sinal CJ. Chemerin exacerbates glucose intolerance in mouse models of obesity and diabetes. Endocrinology. 2010 May;151(5):1998-2007. [191] Liu R, Han Y, Huang C, Hou M, Cheng R, Wang S, et al. Adipocyte-derived chemerin rescues lipid overload-induced cardiac dysfunction. iScience. 2023 Mar 24;26(4):106495. [192] Chen D, Zhang Y, Yidilisi A, Xu Y, Dong Q, Jiang J. Causal Associations Between Circulating Adipokines and Cardiovascular Disease: A Mendelian Randomization Study. J Clin Endocrinol Metab. 2022 May 17;107(6):e2572-e2580. [193] Yi W, Sun Y, Gao E, Wei X, Lau WB, Zheng Q, et al. Reduced cardioprotective action of adiponectin in high-fat diet-induced type II diabetic mice and its underlying mechanisms. Antioxid Redox Signal. 2011 Oct 1;15(7):1779-88. [194] Teoh H, Strauss MH, Szmitko PE, Verma S. Adiponectin and myocardial infarction: A paradox or a paradigm? Eur Heart J. 2006 Oct;27(19):2266-8. [195] Kizer JR, Benkeser D, Arnold AM, Mukamal KJ, Ix JH, Zieman SJ, et al. Associations of total and high-molecular-weight adiponectin with all-cause and cardiovascular mortality in older persons: the Cardiovascular Health Study. Circulation. 2012 Dec 18;126(25):2951-61. [196] Fasshauer M, Paschke R, Stumvoll M. Adiponectin, obesity, and cardiovascular disease. Biochimie. 2004 Nov;86(11):779-84. [197] Sattar N, Nelson SM. Adiponectin, diabetes, and coronary heart disease in older persons: unraveling the paradox. J Clin Endocrinol Metab. 2008 Sep;93(9):3299-301. [198] Kalkman HO. An Explanation for the Adiponectin Paradox. Pharmaceuticals (Basel). 2021 Dec 4;14(12):1266. [199] Liu X, Zhang W, Zhao M, Jia G, Sun R. Effect of atorvastatin treatment on circulating adiponectin: a meta-analysis of randomized controlled trials. Lipids Health Dis. 2019 Dec 23;18(1):228. [200] Sasso FC, Pafundi PC, Marfella R, Calabr P, Piscione F, Furbatto F, et al. Adiponectin and insulin resistance are related to restenosis and overall new PCI in subjects with normal glucose tolerance: the prospective AIRE Study. Cardiovasc Diabetol. 2019 Mar 4;18(1):24. 51 [201] Ktorza A, Bernard C, Parent V, Penicaud L, Froguel P, Lathrop M, et al. Are animal models of diabetes relevant to the study of the genetics of non-insulin-dependent diabetes in humans? Diabetes Metab. 1997 Mar;23 Suppl 2:38-46. [202] Wang B, Chandrasekera PC, Pippin JJ. Leptin- and leptin receptor-deficient rodent models: relevance for human type 2 diabetes. Curr Diabetes Rev. 2014 Mar;10(2):131-45. [203] Shafrir E, Ziv E, Mosthaf L. Nutritionally induced insulin resistance and receptor defect leading to beta-cell failure in animal models. Ann N Y Acad Sci. 1999 Nov 18;892:223-46. [204] Sardu C, D'Onofrio N, Torella M, Portoghese M, Mureddu S, Loreni F, et al. Metformin Therapy Effects on the Expression of Sodium-Glucose Cotransporter 2, Leptin, and SIRT6 Levels in Pericoronary Fat Excised from Pre-Diabetic Patients with Acute Myocardial Infarction. Biomedicines. 2021 Jul 28;9(8):904. [205] Sardu C, Massetti M, Testa N, Martino LD, Castellano G, Turriziani F, et al. Effects of Sodium-Glucose Transporter 2 Inhibitors (SGLT2-I) in Patients With Ischemic Heart Disease (IHD) Treated by Coronary Artery Bypass Grafting via MiECC: Inflammatory Burden, and Clinical Outcomes at 5 Years of Follow-Up. Front Pharmacol. 2021 Nov 15;12:777083. [206] Laurikka A, Vuolteenaho K, Toikkanen V, Rinne T, Leppänen T, Tarkka M, et al. Adipocytokine resistin correlates with oxidative stress and myocardial injury in patients undergoing cardiac surgery. Eur J Cardiothorac Surg. 2014 Oct;46(4):729-36. [207] Lau CH, Muniandy S. Novel adiponectin-resistin (AR) and insulin resistance (IRAR) indexes are

useful integrated diagnostic biomarkers for insulin resistance, type 2 diabetes and metabolic syndrome: a case control study. Cardiovasc Diabetol. 2011 Jan 21;10:8. [208] Habib SS, Al-Khlaiwi T, Butt MA, Habib SM, Al-Khliwi H, Al-Regaiey K. Novel Adiponectin-Resistin Indices and Ratios Predict Increased Cardiovascular Risk in Patients with Type 2 Diabetes Mellitus. J Saudi Heart Assoc. 2023 Apr 8;35(1):59-65. [209] Wang T, Li T, Niu X, Hu L, Cheng J, Guo D, et al. ADSC-derived exosomes attenuate myocardial infarction injury by promoting miR-205-mediated cardiac angiogenesis. Biol Direct. 2023 Feb 27;18(1):6. [210] Comella K, Parcero J, Bansal H, Perez J, Lopez J, Agrawal A, et al. Effects of the intramyocardial implantation of stromal vascular fraction in patients with chron5ic2 ischemic cardiomyopathy. J Transl Med. 2016 Jun 2;14(1):158. [211] Chen CF, Hu CC, Wu CT, Wu HH, Chang CS, Hung YP, et al. Treatment of knee osteoarthritis with intra-articular injection of allogeneic adipose-derived stem cells (ADSCs) ELIXCYTE®: a phase I/II, randomized, active-control, single-blind, multiple-center clinical trial. Stem Cell Res Ther. 2021 Oct 30;12(1):562. [212] Bobi J, Solanes N, Fernnndez-Jiménez R, Galnn-Arriola C, Dantas AP, Fernnndez-Friera L, et al. Intracoronary Administration of Allogeneic Adipose Tissue- Derived Mesenchymal Stem Cells Improves Myocardial Perfusion But Not Left Ventricle Function, in a Translational Model of Acute Myocardial Infarction. J Am Heart Assoc. 2017 May 3;6(5):e005771. [213] Sans-Roselló J, Casals G, Rossello X, Gonznlez de la Presa B, Vila M, Duran- Cambra A, et al. Prognostic value of plasma apelin concentrations at admission in patients with ST-segment elevation acute myocardial infarction. Clin Biochem. 2017 Apr;50(6):279-284. [214] Rastaldo R, Cappello S, Folino A, Losano G. Effect of apelin-apelin receptor system in postischaemic myocardial protection: a pharmacological postconditioning tool? Antioxid Redox Signal. 2011 Mar 1;14(5):909-22. [215] Chen Z, Wu D, Li L, Chen L. Apelin/APJ System: A Novel Therapeutic Target for Myocardial Ischemia/Reperfusion Injury. DNA Cell Biol. 2016 Dec;35(12):766-775. [216] Chapman FA, Maguire JJ, Newby DE, Davenport AP, Dhaun N. Targeting the apelin system for the treatment of cardiovascular diseases. Cardiovasc Res. 2023 Dec 30;119(17):2683-2696. [217] Wu D, Xie F, Xiao L, Feng F, Huang S, He L, et al. Caveolin-1-Autophagy Pathway Mediated Cardiomyocyte Hypertrophy Induced by Apelin-13. DNA Cell Biol. 2017 Aug;36(8):611-618.

522 523 524 525 526 527 528 529 530 531 532 533 534 535 536 537 538 539 540 541 542 543 544 545 546 547 548 549 550 551 552 553 554 555 556 557 558 559 560 561 562 563 564 565 566 567 568 569 570 571 572 573 574 575 576 577 578 579 580 581 582 583 584 585 586 587 588 589 590 591 592 593 594 595 596 597 598 599 600 601 602 603 604 605 606 607 608 609 610 611 612 613 614 615 616 617 618 619 620 621 622 623 624 625 626 627 628 629 630 631 632 633 634 635 636 637 638 639 640 641 642 643 644 645 646 647 648 649 650 651 652 653 654 655 656 657 658 659 660 661 675 676 677 678 679 680 681 682 683 684 685 686 687 688 689 690 691 692 693 694 695 696 697 698 699 700 701 702 703 704 705 706 707 708 709 710 711 712 713 714 715 716 717 718 719 720 721 722 723 724 725 726 727 728 729 730 731 732 733 734 735 736 737 738 739 740 741 742 743 744 745 746 747 748 749 750 751 752 753 754 755 756 757 758 759 760 761 762 763 764 765 766 767 768 769 770 771 772 662 9 10 11 12 27 28 29

sources:

1 59 words / 1% - Internet from 25-Jun-2020 12:00AM
open.library.ubc.ca

2 24 words / < 1% match - from 22-Apr-2024 12:00AM
WWW.MDPI.COM

3 18 words / < 1% match - from 18-Apr-2024 12:00AM
WWW.MDPI.COM

4 16 words / < 1% match - from 21-Oct-2023 12:00AM
www.mdpi.com

5 16 words / < 1% match - Internet from 01-Jan-2020 12:00AM
www.mdpi.com

6 10 words / < 1% match - from 01-Dec-2023 12:00AM
WWW.MDPI.COM

7 8 words / < 1% match - from 24-Jan-2024 12:00AM
www.mdpi.com

8 15 words / < 1% match - from 19-Mar-2024 12:00AM
www.frontiersin.org

9 14 words / < 1% match - from 06-Oct-2023 12:00AM
www.frontiersin.org

10 11 words / < 1% match - from 08-Jul-2024 12:00AM
www.frontiersin.org

11 10 words / < 1% match - from 08-Jul-2024 12:00AM
www.frontiersin.org

12 8 words / < 1% match - from 31-Oct-2023 12:00AM
www.frontiersin.org

13 8 words / < 1% match - from 01-Jun-2023 12:00AM
www.frontiersin.org

14 8 words / < 1% match - from 08-Jul-2024 12:00AM
www.frontiersin.org

15 8 words / < 1% match - Internet from 25-Nov-2020 12:00AM
www.frontiersin.org

16 21 words / < 1% match - Internet
Juntao Yang, Jiedong Zhou, Hanxuan Liu, Jinjin Hao, Songqing Hu, Peipei Zhang, Haowei Wu, Yefei Gao, Weiliang Tang. "Blood lipid levels mediating the effects of sex hormone-binding globulin on coronary heart disease: Mendelian randomization and mediation analysis", Scientific Reports

17 16 words / < 1% match - Internet
Yafeng Wang, Lu Zhou, Wating Su, Fengnan Huang, Yuan Zhang, Zhong-yuan Xia, Zhengyuan Xia, Shaoqing Lei. "Selective Inhibition of PKC2 Restores Ischemic Postconditioning-Mediated Cardioprotection by Modulating Autophagy in Diabetic Rats", Journal of Diabetes Research

18 12 words / < 1% match - Internet
Cia-Hin Lau, Sekaran Muniandy. "Novel adiponectin-resistin (AR) and insulin resistance (IR) indexes are useful integrated diagnostic biomarkers for insulin resistance, type 2 diabetes and metabolic syndrome: a case control study", Cardiovascular Diabetology

19 9 words / < 1% match - Internet from 02-Dec-2009 12:00AM
www.ncbi.nlm.nih.gov

20 9 words / < 1% match - Internet
Roland Akhigbe, Ayodeji Ajayi. "The impact of reactive oxygen species in the development of cardiometabolic disorders: a review", Lipids in Health and Disease

21 14 words / < 1% match - Internet from 18-Nov-2022 12:00AM
pubmed.ncbi.nlm.nih.gov

22 10 words / < 1% match - from 25-Oct-2024 12:00AM
pubmed.ncbi.nlm.nih.gov

23 8 words / < 1% match - from 13-Jul-2023 12:00AM
pubmed.ncbi.nlm.nih.gov

24 8 words / < 1% match - from 19-Feb-2024 12:00AM
pubmed.ncbi.nlm.nih.gov

25 38 words / < 1% match - Internet from 13-Mar-2022 12:00AM
www.pure.ed.ac.uk

26 35 words / < 1% match - Internet from 26-May-2016 12:00AM
orbilu.uni.lu

27 12 words / < 1% match - Internet from 25-Nov-2018 12:00AM
www.nature.com

28 10 words / < 1% match - from 18-Jul-2024 12:00AM
www.nature.com

29 9 words / < 1% match - Internet from 18-Aug-2022 12:00AM
www.nature.com

30 27 words / < 1% match - Internet from 12-Oct-2022 12:00AM
mdpi-res.com

31 17 words / < 1% match - from 02-Aug-2024 12:00AM
cardiab.biomedcentral.com

32 9 words / < 1% match - Internet from 09-Mar-2020 12:00AM
cardiab.biomedcentral.com

33 25 words / < 1% match - ProQuest
Ameka, Magdalene Khang'ai. "The Role of FGF21 in Regulating Energy Homeostasis.", The University of Iowa, 2018

34 25 words / < 1% match - Internet from 06-Oct-2022 12:00AM
era.ed.ac.uk

35 14 words / < 1% match - from 17-Mar-2023 12:00AM
link.springer.com

36 11 words / < 1% match - Internet from 25-Feb-2019 12:00AM
link.springer.com

37 15 words / < 1% match - from 31-May-2024 12:00AM
www2.mdpi.com

38 10 words / < 1% match - from 22-Dec-2023 12:00AM
www2.mdpi.com

39 15 words / < 1% match - Internet from 17-Dec-2019 12:00AM
www.spandidos-publications.com

40 9 words / < 1% match - from 11-May-2024 12:00AM
www.spandidos-publications.com

41 21 words / < 1% match - from 24-Feb-2024 12:00AM
vdoc.pub

42 20 words / < 1% match - Crossref
Nadtochiy, S.M.. "Mediterranean diet and cardioprotection: The role of nitrite, polyunsaturated fatty acids, and polyphenols", Nutrition, 201107/08

43 19 words / < 1% match - Crossref
Tiangui Yang, Daqing Zhang. "Research Progress on the Effects of Novel Hypoglycemic Drugs in Diabetes Combined with Myocardial Ischemia/Reperfusion Injury", Ageing Research Reviews, 2023

44 19 words / < 1% match - from 05-Sep-2023 12:00AM
dokumen.pub

45 9 words / < 1% match - from 07-Feb-2024 12:00AM
academic.oup.com

46 8 words / < 1% match - Internet from 23-Oct-2022 12:00AM
academic.oup.com

47 16 words / < 1% match - Internet from 03-Mar-2022 12:00AM
research-repository.griffith.edu.au

48 15 words / < 1% match - Internet from 05-May-2016 12:00AM
circres.ahajournals.org

49 15 words / < 1% match - from 13-Nov-2023 12:00AM
www.degruyter.com

50 13 words / < 1% match - from 03-Aug-2023 12:00AM
jpet.aspetjournals.org

51 13 words / < 1% match - Internet from 21-Sep-2020 12:00AM
www.researchsquare.com

52 12 words / < 1% match - Crossref
Chiara Tersigni, Fiorella Di Nicuolo, Silvia D’Ippolito, Manuela Veglia, Mario Castellucci, Nicoletta Di Simone. "Adipokines: New Emerging Roles in Fertility and Reproduction", Obstetrical & Gynecological Survey, 2011

53 12 words / < 1% match - Crossref
Liang Ge, Yin Cai, Fan Ying, Hao Liu, Dengwen Zhang, Yanjing He, Lei Pang, Dan Yan, Aimin Xu, Haichun Ma, Zhengyuan Xia. "miR-181c-5p Exacerbates Hypoxia/Reoxygenation-Induced Cardiomyocyte Apoptosis via Targeting PTPN4", Oxidative Medicine and Cellular Longevity, 2019

---

54 12 words / < 1% match - Crossref
Serdar Farhan, Björn Redfors, Akiko Maehara, Thomas McAndrew et al. "Relationship between insulin resistance, coronary plaque, and clinical outcomes in patients with acute coronary syndromes: an analysis from the PROSPECT study", Cardiovascular Diabetology, 2021

---

55 12 words / < 1% match - Crossref
Shaghayegh Hemat Jouy, Sukrutha Mohan, Giorgia Scichilone, Amro Mostafa, Abeer M. Mahmoud. "Adipokines in the Crosstalk between Adipose Tissues and Other Organs: Implications in Cardiometabolic Diseases", Biomedicines, 2024

---

56 12 words / < 1% match - Crossref
Zeina Harhous, George W. Booz, Michel Ovize, Gabriel Bidaux, Mazen Kurdi. "An Update on the Multifaceted Roles of STAT3 in the Heart", Frontiers in Cardiovascular Medicine, 2019

---

57 12 words / < 1% match - Internet
Ferdinandy, Peter, Hausenloy, Derek J., Heusch, Gerd, Baxter, Gary Francis, Schulz, Rainer. "Interaction of risk factors, comorbidities, and comedications with ischemia/reperfusion injury and cardioprotection by preconditioning, postconditioning, and remote conditioning", American Society for Pharmacology and Experimental Therapeutics, 2014

---

58 11 words / < 1% match - Crossref
Handbook of Experimental Pharmacology, 2016.

---

59 11 words / < 1% match - Internet from 11-May-2021 12:00AM
www.hindawi.com

---

60 10 words / < 1% match - Crossref
"Metabolic Syndrome and Neurological Disorders", Wiley, 2013

---

61 10 words / < 1% match - Crossref
Chuanbin Chen, Chuanghong Lu, Dewei He, Na Na, Yunjiao Wu, Zuchun Luo, Feng Huang. "Inhibition of HMGB1 alleviates myocardial ischemia/reperfusion injury in diabetic mice via suppressing autophagy", Microvascular Research, 2021

---

62 10 words / < 1% match - Publications
Neeraj Mishra, Sumel Ashique, Farid Arshad, Garg Ashish. "Synbiotics in Metabolic Disorders - Mechanisms, Therapeutic Potential, and Future Perspectives", CRC Press, 2024

---

63 10 words / < 1% match - Crossref
Pin-Fang Kang, Wen-Juan Wu, Yang Tang, Ling Xuan, Su-Dong Guan, Bi Tang, Heng Zhang, Qin Gao, Hong-Ju Wang. "Activation of ALDH2 with Low Concentration of Ethanol Attenuates Myocardial Ischemia/Reperfusion Injury in Diabetes Rat Model", Oxidative Medicine and Cellular Longevity, 2016

---

64 10 words / < 1% match - Crossref
Shan, L.. "Disruption of Rac1 signaling reduces ischemia-reperfusion injury in the diabetic heart by inhibiting calpain", Free Radical Biology and Medicine, 20101201

65 10 words / < 1% match - Crossref
Shen, Zi-Ying, Qian Sun, Zhong-Yuan Xia, Qing-Tao Meng, Shao-Qing Lei, Bo Zhao, Ling-Hua Tang, Rui Xue, and Rong Chen. "Overexpression of DJ-1 reduces oxidative stress and attenuates hypoxia/reoxygenation injury in NRK-52E cells exposed to high glucose", International Journal of Molecular Medicine, 2016.

66 10 words / < 1% match - Crossref
Tao Li, Shan-Shan Yu, Chang-Yu Zhou, Ke Wang, Ying-Chun Wan. "RETRACTED ARTICLE: MicroRNA-206 inhibition and activation of the AMPK/Nampt signalling pathway enhance sevoflurane post-conditioning-induced amelioration of myocardial ischaemia/reperfusion injury", Journal of Drug Targeting, 2019

67 10 words / < 1% match - Internet from 27-Aug-2022 12:00AM
portlandpress.com

68 10 words / < 1% match - Internet from 26-Sep-2018 12:00AM
www.labome.org

69 10 words / < 1% match - Internet from 19-Sep-2021 12:00AM
www.sciencegate.app

70 9 words / < 1% match - Crossref
Celestino Sardu, Gorizio Pieretti, Nunzia D'Onofrio, Feliciano Ciccarelli et al. "Inflammatory Cytokines and SIRT1 Levels in Subcutaneous Abdominal Fat: Relationship With Cardiac Performance in Overweight Pre-diabetics Patients", Frontiers in Physiology, 2018

71 9 words / < 1% match - Publications
Christian Weber, Oliver Soehnlein. "Atherosclerosis - Treatment and Prevention", Pan Stanford, 2019

72 9 words / < 1% match - Crossref
Dongyun Zhang, Qun Wang, Xunbin Qiu, Yiguan Chen, Xiaoli Yang, Yujian Guan. "Remifentanil protects heart from myocardial ischaemia/reperfusion (I/R) injury via miR-206-3p/TLR4/NF-κB signalling axis", Journal of Pharmacy and Pharmacology, 2022

73 9 words / < 1% match - Crossref
Han Feng, Hao Shen, Matthew J. Robeson, Yue-Han Wu et al. "MG53 E3 Ligase–Dead Mutant Protects Diabetic Hearts From Acute Ischemic/Reperfusion Injury and Ameliorates Diet-Induced Cardiometabolic Damage", Diabetes, 2022

74 9 words / < 1% match - Crossref
Jan Bilski, Agata Schramm-Luc, Marian Szczepanik, Agnieszka Irena Mazur-Biały et al. "Adipokines in Rheumatoid Arthritis: Emerging Biomarkers and Therapeutic Targets", Biomedicines, 2023

75 9 words / < 1% match - Crossref
Kerstin Boengler, Chantal Eickelmann, Petra Kleinbongard. "Mitochondrial Kinase Signaling for Cardioprotection", International Journal of Molecular Sciences, 2024

76 9 words / < 1% match - Crossref
M. Collino, M. Aragno, S. Castiglia, C. Tomasinelli, C. Thiemermann, G. Boccuzzi, R. Fantozzi. "Insulin Reduces Cerebral Ischemia/Reperfusion Injury in the Hippocampus of Diabetic Rats: A Role for Glycogen Synthase Kinase-3", Diabetes, 2008

77 9 words / < 1% match - Crossref
Shaoqing Lei, Wating Su, Zhong-Yuan Xia, Yafeng Wang, Lu Zhou, Shigang Qiao, Bo Zhao, Zhengyuan Xia, Michael G. Irwin. "Hyperglycemia-Induced Oxidative Stress Abrogates Remifentanil Preconditioning-Mediated Cardioprotection in Diabetic Rats by Impairing Caveolin-3-Modulated PI3K/Akt and JAK2/STAT3 Signaling", Oxidative Medicine and Cellular Longevity, 2019

78 9 words / < 1% match - Crossref
Ziegelmeier, M.. "Adipokines influencing metabolic and cardiovascular disease are differentially regulated in maintenance hemodialysis", Metabolism, 200810

79 9 words / < 1% match - from 14-Sep-2024 12:00AM
ebin.pub

80 9 words / < 1% match - Internet from 22-Aug-2022 12:00AM
idoc.pub

81 9 words / < 1% match - Internet from 29-Sep-2022 12:00AM
joe.bioscientifica.com

82 9 words / < 1% match - Internet from 12-Sep-2015 12:00AM
www.420magazine.com

83 9 words / < 1% match - Internet from 13-Feb-2014 12:00AM
www.acnut.com

84 9 words / < 1% match - Internet from 28-Feb-2023 12:00AM
www.researchgate.net

85 9 words / < 1% match - from 23-Feb-2024 12:00AM
www.Scirp.org

86 9 words / < 1% match - from 28-Jul-2024 12:00AM
www.springermedizin.de

87 9 words / < 1% match - Internet from 05-Sep-2018 12:00AM
www.tandfonline.com

88 8 words / < 1% match - Crossref
Andreas Mitsis, Elina Khattab, Michael Myrianthefs, Stergios Tzikas et al. "Chemerin in the Spotlight: Revealing Its Multifaceted Role in Acute Myocardial Infarction", Biomedicines, 2024

89 8 words / < 1% match - Crossref
Cong Chen, Jie Wang, Shan Zhang, Xueying Zhu, Jun Hu, Chao Liu, Lanchun Liu. "Epigenetic regulation of diverse regulated cell death modalities in cardiovascular disease: Insights into necroptosis, pyroptosis, ferroptosis, and cuproptosis", Redox Biology, 2024

90 8 words / < 1% match - Publications
Giuseppe Mancia, Guido Grassi, Konstantinos P. Tsioufis, Anna F. Dominiczak, Enrico Agabiti Rosei. "Manual of Hypertension of the European Society of Hypertension", CRC Press, 2019

91 8 words / < 1% match - Crossref
Huang Xin, Wang Jianan. "UP-REGULATION OF ENDOGENOUS LEPTIN IMPROVES HUMAN MESENCHYMAL STEM CELL SURVIVAL ABILITY IN VITRO AND THIS CELLS PROTECT FATAL CARDIAL MYOCYTES FROM APOPTOSIS", Heart, 2012

92 8 words / < 1% match - Publications
Isaias Dichi, José Wander Breganó, Andréa Name Colado Simão, Rubens Cecchini. "Role of Oxidative Stress in Chronic Diseases", CRC Press, 2014

93 8 words / < 1% match - Crossref
Jared Tur, Sachin L. Badole, Ravikumar Manickam, Kalyan C. Chapalamadugu et al. " ", Journal of Pharmacology and Experimental Therapeutics, 2022

94 8 words / < 1% match - Crossref
Jinli Wang, Li Fan. "Cardioprotective effect of Perakine against myocardial ischemia-reperfusion injury of type-2 diabetic rat in Langendorff-Perfused Rat Hearts: Role of TLR4/NF-kB signalling pathway", Folia Morphologica, 2024

95 8 words / < 1% match - ProQuest
Jones, Holly Mei. "The Effect of Patterns and Distributions of Physical Activity on Blood Glucose Control in Individuals with Type 2 Diabetes Mellitus: An Exploratory Study", University of Exeter (United Kingdom), 2023

96 8 words / < 1% match - ProQuest
Kanoriya, Dharmendra. "Correlation of Retinol Binding Protein 4 (RBP 4) and Leptin Levels in Crevicular Fluid and Serum in Chronic Periodontitis Subjects with and Without Obesity", Rajiv Gandhi University of Health Sciences (India), 2023

97 8 words / < 1% match - Publications
Leo M.L. Nollet, Javed Ahamad. "Bioactive Compounds of Edible Oils and Fats - Health Benefits, Risks, and Analysis", CRC Press, 2024

98 8 words / < 1% match - Publications
Margaret Rees, Mahantesh Karoshi, Louis Keith. "Obesity and Pregnancy", CRC Press, 2024

99 8 words / < 1% match - Publications
Nilanjana Maulik. "Cardiovascular Diseases - Nutritional and Therapeutic Interventions", CRC Press, 2019

100 8 words / < 1% match - Crossref

Paula Müller, Heiko Lemcke, Robert David. "Stem Cell Therapy in Heart Diseases – Cell Types, Mechanisms and Improvement Strategies", Cellular Physiology and Biochemistry, 2018

101

8 words / < 1% match - Crossref
Penna, Claudia, Maria-Giulia Perrelli, and Pasquale Pagliaro. "Mitochondrial Pathways, Permeability Transition Pore and Redox Signaling in Cardioprotection: Therapeutic Implications", Antioxidants & Redox Signaling, 2012.

102

8 words / < 1% match - Crossref
Qin Huang, Hao Tian, Liqun Tian, Xiaoshuai Zhao, Lu Li, Yuxi Zhang, Zhen Qiu, Shaoqing Lei, Zhongyuan Xia. "Inhibiting Rev-erbα-mediated ferroptosis alleviates susceptibility to myocardial ischemia-reperfusion injury in type 2 diabetes", Free Radical Biology and Medicine, 2023

103

8 words / < 1% match - Crossref
Ruimin Liu, Yinying Han, Chenglong Huang, Mengqian Hou, Rui Chen, Shujin Wang, Xi Li, Jie Tian. "Adipocyte-derived chemerin rescues lipid overload-induced cardiac dysfunction", iScience, 2023

104

8 words / < 1% match - Crossref
Shiyi Lin, Xin Li, Jiabei Zhang, Yuyang Zhang. "Omentin-1: Protective impact on ischemic stroke via ameliorating atherosclerosis", Clinica Chimica Acta, 2021

105

8 words / < 1% match - Crossref
Wang, Yanwen. "Obesity and Related Disorders", Functional Food Product Development Smith/Functional Food Product Development, 2010.

106

8 words / < 1% match - Crossref
Zhao V. Wang, Philipp E. Scherer. "Adiponectin, the past two decades", Journal of Molecular Cell Biology, 2016

107

8 words / < 1% match - Crossref
Zhen Huang, Lele Wu, Linxi Chen. "Apelin/APJ system: A novel potential therapy target for kidney disease", Journal of Cellular Physiology, 2018

108

8 words / < 1% match - Crossref
Zhijun Meng, Bin Liang, Yalin Wu, Caihong Liu et al. "Hypoadiponectinemia-induced upregulation of microRNA449b downregulating Nrf-1 aggravates cardiac ischemia-reperfusion injury in diabetic mice", Journal of Molecular and Cellular Cardiology, 2023

109

8 words / < 1% match - Internet
Yue Xie, Xiaozhen Quan, Xuezhou Yang. "Raised levels of chemerin in women with preeclampsia: A meta-analysis", Association of Basic Medical Sciences of Federation of Bosnia and Herzegovina, 2023

110

8 words / < 1% match - from 26-Mar-2024 12:00AM
era.library.ualberta.ca

111

8 words / < 1% match - Internet from 10-Jan-2015 12:00AM
jddtonline.info

112 8 words / < 1% match - from 01-Aug-2024 12:00AM
peerj.com

113 8 words / < 1% match - from 24-Oct-2024 12:00AM
pmc.ncbi.nlm.nih.gov

114 8 words / < 1% match - Internet from 10-Nov-2022 12:00AM
thno.org

115 8 words / < 1% match - from 01-Jan-2024 12:00AM
watermark.silverchair.com

116 8 words / < 1% match - Internet from 31-Jul-2018 12:00AM
www.jove.com

117 8 words / < 1% match - Internet from 06-Aug-2022 12:00AM
www.scielo.br

118 8 words / < 1% match - Internet from 07-Feb-2022 12:00AM
www.science.gov

119 8 words / < 1% match - Internet from 24-Feb-2015 12:00AM
www.slideshare.net
