# Supplementary material for: Adipokines and their potential impacts on susceptibility to myocardial ischemia/reperfusion injury in diabetes
Source: Lipids Health Dis. 2024 Nov 13;23:372. doi: 10.1186/s12944-024-02357-w (PMC11558907; doi:10.1186/s12944-024-02357-w)
Supplement: Supplementary file 2 — Supplementary Material 2. [file 12944_2024_2357_MOESM2_ESM.pdf]

Vanscholar Editors Co. Ltd

Address: 307-2280 Wesbrook Mall
Vancouver, BC, V6T 2K3, Canada
Phone: +1 778-6866948 Fax: +1 604-2229501

Web: www.vanscholareditors.com

Email: infor@vanscholareditors.com

Editorial Certification

This is to certify that the manuscript entitled " **Adipokines and their potential impacts on susceptibility to myocardial ischemia/reperfusion injury in diabetes**" was edited for English language usage, grammar, spelling and punctuation by one senior native English-speaking editors at Vanscholar Editing Service at the level of advanced service. The editors focused in correcting improper language and rephrasing awkward sentences, using their scientific training to point out passages that were confusing or vague. Every effort has been made to ensure that neither the research content nor the authors' intention were altered in any way during the editing process.

Documents receiving this certification should be English-ready for publication; however, please note that the authors have the ability to accept or reject our suggestions and changes. If you have any question or concerns over this edited document, please contact Vanscholar Editing Service at **infor@vanscholareditors.com**

Manuscript title: Adipokines and their potential impacts on susceptibility to myocardial ischemia/reperfusion injury in diabetes

Authors: Ronghui Han, Hemeng Huang, Jianyu Zhu, Xiaogao Jin, Yongyan Wang, Youhua Xu, Zhengyuan Xia

Certificate Number: VE2024-BS-0907A

This certificate, issued on October 30, 2024, may be verified at www.vanscholareditors.com
